# Chimeric Antigen Receptor T Cells as Living Therapeutics Targeting Senescence and Age-Related Diseases

**DOI:** 10.34133/research.1326

**Published:** 2026-06-12

**Authors:** Qingyi Shao, Danlei Chen, Wenxia Shao, Qing Ye

**Affiliations:** ^1^ Department of Laboratory Medicine, Children’s Hospital, Zhejiang University School of Medicine, National Clinical Research Center for Children and Adolescents’ Health and Diseases, Hangzhou 310052, China.; ^2^ Department of Laboratory Medicine, Affiliated Hangzhou First People’s Hospital, School of Medicine, Westlake University, Hangzhou 310006, China.

## Abstract

The aging of the global population exacerbates the burden of age-related diseases; however, therapies that can intervene in fundamental aging processes are lacking. Senescent cells drive chronic inflammation and multitissue dysfunction through the secretion of proinflammatory and profibrotic senescence-associated secretory phenotype cells, making them emerging therapeutic targets. Although first-generation senolytic drugs have entered clinical trials, they face limitations such as insufficient targeting specificity and transient efficacy. The success of chimeric antigen receptor T cell (CAR T cell) therapy in cancer immunotherapy has validated its precision clearance capabilities as a “living drug”. This review systematically elaborates on the paradigm shift of extending CAR T cell therapy to aging medicine, from the discovery and validation of surface targets on senescent cells to a CAR engineering design tailored to the senescent microenvironment and from evidence of reversing fibrosis and improving metabolic function in preclinical models to the challenges of specificity, safety, and manufacturing faced in clinical translation. Finally, future directions for integrating technologies such as mRNA delivery and artificial intelligence are envisioned in this article, which proposes that CAR T cell therapy may drive the evolution of medicine from “treating single diseases” to “intervening in shared aging processes”, offering transformative strategies to achieve healthy aging.

## Introduction

In the 21st century, the global population structure is undergoing a profound shift toward aging, accompanied by a sharp increase in the incidence of age-related diseases, posing severe challenges to socioeconomic and healthcare systems [1]. Organismal aging, as a fundamental biological phenomenon, underlies the development and exacerbation of multiple age-related pathologies, including Alzheimer’s disease, cardiovascular conditions, osteoarthritis (OA), and pulmonary fibrosis [2]. However, most approved drugs target symptoms or single pathways and fail to directly intervene in senescent cells or reverse/delay the underlying drivers of aging. Cellular senescence, defined as an irreversible halt in cell division, is essential for normal tissue regeneration and cancer prevention [3]. However, with aging or chronic stress, senescent cells abnormally accumulate in tissues. Characterized by permanent withdrawal from the cell cycle and evasion of programmed cell death, these cells nonetheless maintain robust metabolic function. Their persistent secretion of proinflammatory cytokines, matrix-degrading enzymes, and mitogenic signals constitutes what is known as the senescence-associated secretory phenotype (SASP) [4,5]. In addition to being governed primarily by the DNA damage response (DDR), the SASP is modulated through an intricate network of signaling cascades encompassing the p53–p21, p16–Rb, nuclear factor κB (NF-κB), mechanistic target of rapamycin (mTOR), and p38 mitogen-activated protein kinase (MAPK) axes [6]. These pathways collectively induce and modulate the secretory phenotype, making it highly dynamic and variable depending on the type of senescence and the cellular context [4,7]. The SASP creates a disruptive microenvironment, triggering chronic low-grade inflammation (inflammaging), impairing neighboring cell function, disrupting tissue homeostasis, and directly promoting the pathological progression of numerous age-associated disorders [7–10]. Therefore, targeted elimination of aging cells with high specificity or inhibition of their harmful secretions has become important as a next-generation weapon against biological aging [11]. First-generation senolytics such as navitoclax and dasatinib combined with quercetin, which clear senescent cells by interfering with their antiapoptotic pathways, have demonstrated preliminary efficacy in both preclinical models and early-phase human studies to improve various aging phenotypes [12,13]. However, small-molecule drugs face challenges such as pharmacokinetic limitations (uneven tissue distribution and poor blood–brain barrier penetration), efficacy fluctuations due to intermittent dosing, and potential off-target toxicity [14]. Senomorphics focus on modulating the SASP while maintaining cellular survival but require long-term administration and fail to eradicate the latent risks posed by senescent cells [15]. These limitations have spurred the need for more precise, durable, and controllable therapeutic strategies. Concurrently, breakthroughs have been made in adoptive cell immunotherapy [16]. Chimeric antigen receptor (CAR)-engineered T cell therapy, whereby a patient’s own immune cells are retrofitted with synthetic receptors for precise tumor recognition, has emerged as a transformative modality in the oncological management of hematologic origin [17–20]. As “living drugs”, CAR T cells possess unique advantages, including long-term survival, expansion in vivo, and precise targeting of diseased cells [21–25]. This success has inspired researchers to explore whether this powerful immunotherapy platform can be repurposed to identify and eliminate another type of “diseased” cell—senescent cells. This review systematically examines the scientific foundation, technological evolution, and application prospects of extending CAR T cell therapy from cancer immunotherapy to senescence therapy [26]. We first dissect the molecular characteristics of senescent cells and their pathological contributions and then detail how CAR T cell engineering strategies can be adapted to target senescence. Next, we summarize key preclinical evidence of CAR T cell interventions directed at senescent cell populations, particularly their dual “clearance–repair” effects in reversing core pathologies such as fibrosis. We also address critical challenges in clinical translation, including specificity, safety, and manufacturing. Finally, we envision future integration with emerging technologies and the potential of CAR T cells as precision antiaging therapies. All CAR-T-cell-based antiaging research discussed herein remains in the preclinical stage and has not yet received approval from any regulatory authority [27,28].

## Molecular Landscape of Senescent Cells and Disease Association

**Fig. 1.**
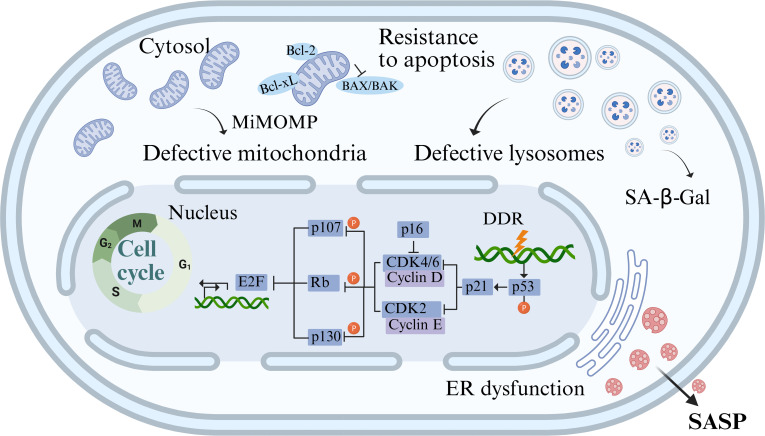
Characteristics of senescent cells. ER, endoplasmic reticulum; Bcl-2, B cell lymphoma-2; BAX, Bcl-2-associated X protein; BAK, Bcl-2 homologous antagonist/killer; miMOMP, minority mitochondrial outer membrane permeabilization.

The aging process is a major factor in the regulation of embryonic development, cellular reprogramming, wound healing, and tumor suppression [29–32]. However, cellular senescence is also a key factor in the initiation and progression of age-dependent diseases such as Alzheimer’s disease [33–35], osteoporosis [36,37], atherosclerosis [38–40], and macular degeneration [41,42]. Typically, senescent cells exhibit morphological features such as increased cell volume and irregular nuclear size [43–47], accompanied by organelle dysfunction, such as lysosomal defects, mitochondrial defects, and endoplasmic reticulum dysfunction (Fig. 1) [4,48]. In terms of induction mechanisms, cellular senescence can be classified into replicative senescence, oncogene-induced senescence, therapy-induced senescence, mitochondrial-dysfunction-induced senescence, and immune-induced senescence [4,49]. Replicative senescence is typically associated with telomere shortening, cell cycle arrest, and SASP [50,51], which are caused primarily by gradual telomere shortening and repeated cell division. Oncogene-related RAS/MAPK and phosphatidylinositol 3-kinase/AKT/mTOR pathways drive oncogene-induced senescence, which is often accompanied by oxidative stress and DNA damage [52,53]. Therapy-induced senescence is triggered by chemotherapy, radiotherapy, or other therapeutic interventions to inhibit tumor cell proliferation [54].

Although the triggers are diverse, they ultimately converge on 2 core cell cycle inhibitory pathways: the p53–p21 pathway and the p16^INK4a^ (p16)–Rb pathway (Fig. 1). The p16 protein inhibits the interaction between cyclin-dependent kinase 4/6 (CDK4/6) and cyclin D, maintaining the Rb protein in a prephosphorylated state, thereby inhibiting E2 transcription factor (E2F) activity and impeding G_1_–S phase transition and subsequent cell cycle progression. The DDR activates p53, up-regulates p21, and inhibits the binding of CDK2 to cyclin E, similarly blocking cell cycle progression [55,56]. In addition to growth arrest, a hallmark feature of senescent cells is their highly active SASP. The composition of the SASP is complex and diverse and encompasses the following major categories: inflammatory factors (e.g., interleukin-6 [IL-6] and IL-1α), chemokines (e.g., IL-8 and monocyte chemoattractant protein-1), matrix metalloproteinases (MMPs; e.g., MMP-1/3), and growth factors (e.g., vascular endothelial growth factor and transforming growth factor-β [TGF-β]), among others. Its expression is strongly regulated by multiple intracellular communication pathways, specifically the NF-κB, CCAAT/enhancer-binding protein beta, mTOR, and p38 MAPK kinase cascades [5]. SASP is not entirely harmful; it can play a transient positive role in tissue remodeling following acute injury [5]. However, as senescent cells persist long term and SASP secretion continues, the role of the SASP gradually shifts from its physiological function to its role as a pathological driver. Single-cell transcriptomic analyses have revealed substantial phenotypic diversity among senescent cell populations, with different senescent cell populations displaying diverse cellular senescence characteristics, such as elevated mRNA levels of extracellular-matrix-organizing proteins and antiapoptotic proteins, reduced mRNA levels of oxidative phosphorylation proteins, and increased levels of long noncoding RNAs [57].

**Fig. 2.**
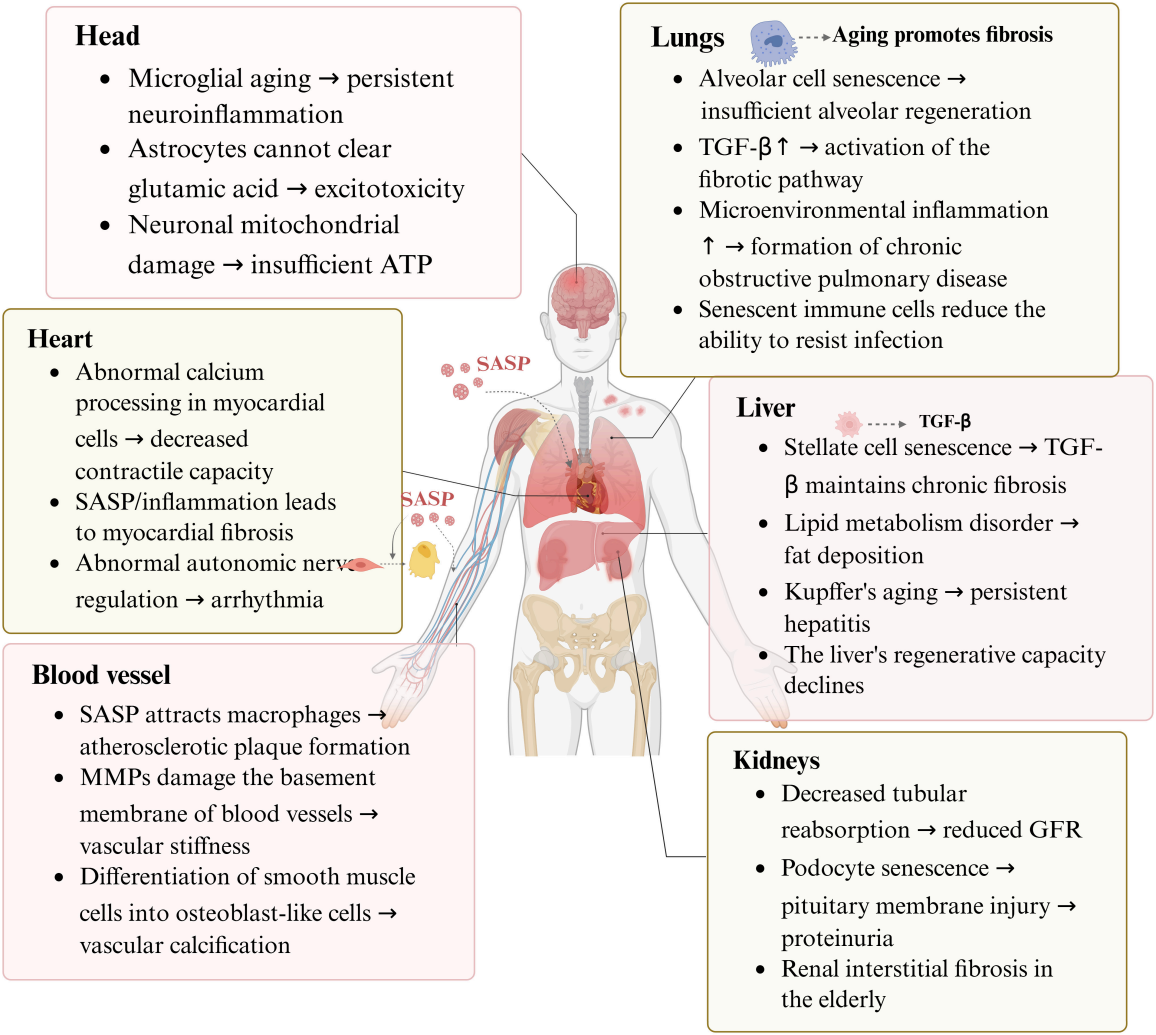
Role of senescent cells in the pathogenesis of age-related diseases. ATP, adenosine triphosphate; GFR, glomerular filtration rate.

In different organs, senescent cells contribute to diseases through specific mechanisms (Fig. 2). In the vasculature, SASP activation from senescent smooth muscle and endothelial cells results in the establishment of a proinflammatory microenvironment. Macrophage recruitment is thereby enhanced, atherosclerotic plaque dynamics are destabilized, and osteoblastic conversion of smooth muscle cells is promoted—collectively resulting in vascular calcific deposition [58–60]. In the lungs, senescent alveolar epithelial cells drive myofibroblast differentiation and exacerbate interstitial lung fibrosis through the secretion of factors such as TGF-β [61]. In the liver, senescent hepatic stellate cells increase TGF-β levels, and lipid metabolism disorders lead to fat deposition, resulting in persistent hepatitis and a decrease in liver regeneration capacity [62,63]. In the nervous system, inflammatory factors released by senescent microglia and astrocytes exacerbate neuroinflammation, promoting the emergence of neurodegenerative diseases such as Alzheimer’s disease [64]. Therefore, senescent cells serve as “common nodes”, linking the fundamental aging process to various age-related diseases, and targeting them offers potential for intervention in multiple diseases.

## CAR T Cell Platform: Engineered Evolution and Senescence-Targeted Modification

### Structural basis and modular design

The primary objective of CAR T cell immunotherapy lies in integrating T cell cytotoxic potency with antibody-like target recognition to achieve precise eradication of pathological cells [65]. The typical CAR structure consists of an extracellular single-chain fragment (scFv), a hinge region that enhances receptor flexibility, a transmembrane domain, a costimulatory domain, and a T cell activation signaling domain (CD3ζ) [21]. Through plasticity designs, such as targeting specific antigens, multitarget strategies to prevent antigen escape, optimized costimulatory domains (CD28 and 4-1BB), and T cell activation signaling domains (CD3ζ), CAR T cell therapy is applied not only in hematological malignancies but also in targeting various diseases and pathological substrates [66,67]. To address challenges in tumor treatment, such as antigen escape and tumor microenvironment suppression, CAR T cell technology has developed highly modular and programmable engineering strategies, which are also applicable to senescence targeting. Immune checkpoint inhibitors (such as programmed cell death protein 1 [PD-1] immune checkpoint blockade [68,69] and negative cytotoxic T-lymphocyte-associated protein-4 regulation [70]) are among the key armor design strategies for combating the immunosuppressive microenvironment that may exist in aging tissues. Safety switches, such as the inducible caspase 9 (iCasp9) suicide gene, can specifically induce CAR T cell apoptosis via small-molecule drug administration as an emergency safeguard against severe adverse events, offering a rapid safety override mechanism [71].

The ultrasound-controlled EchoBack-CAR platform achieves noninvasive remote regulation of CAR expression through the integration of thermosensitive promoters with synthetic-biology-derived positive feedback circuits: Focused-ultrasound-induced local hyperthermia (e.g., 43 °C for 15 min) specifically activates evolutionarily optimized heat-inducible promoters, thereby driving CAR expression; critically, this platform incorporates T cell activation signal-sensing positive feedback loops (e.g., the nuclear factor of activated T cells and NF-κB pathways) that sustain CAR expression following ultrasound triggering, overcoming the transient limitation of conventional inducible systems [72]. This system achieves “echo-like” sustained activation through an ultrasensitive heat-shock promoter combined with a positive feedback loop, demonstrating an approximately 90% tumor volume reduction without off-target toxicity in glioblastoma models; its deep tissue penetration and noninvasive characteristics make it particularly suitable for chronic age-related conditions such as liver fibrosis and natural aging, which require repeated dosing, although temperature sensitivity and equipment dependency remain limitations [73].

Concurrently, researchers developed a red light/far-red light-regulated single-cell encapsulation system utilizing the plant-derived photoreceptor truncated phytochrome A–phycocyanobilin (ΔPhyA-PCB) to achieve bidirectional reversible switching of gene expression: 660-nm red light induces heterodimerization to initiate effector molecule (interferon-γ, IL-6, and anti-CD47 antibody) expression, whereas 730-nm-long red light provides immediate termination [74,75]. The split-CAR (light-inducible CAR) system splits the CAR into 2 components, each of which is fused with a light-inducible dimerization domain. Upon blue light irradiation, the 2 components heterodimerize to reconstitute a functional CAR, enabling precise optogenetic control of CAR T cell activity [74]. Light-inducible CAR T cells can specifically suppress tumors in illuminated areas while reducing cytokine release syndrome; their micrometer-scale precision is suitable for superficial tissues such as skin aging, yet the limited penetration of blue light (<1 mm) limits deep tissue applications.

In addition, orthogonal CRISPR systems have been used to combine the cytosine base editor Nme2Cas9—lacking DNA double-strand break risk—with SpyCas9 nuclease, enabling multiplex gene knockout without viral vectors, including human lymphocyte antigen-A (HLA-A), HLA-B, and class II major histocompatibility complex transactivator deletion, to evade host immune rejection, alongside site-specific CAR gene integration at the T cell receptor α constant locus for efficient generation of universal CAR T cells [76]. Studies have revealed that forkhead box P3 induces distinctive metabolic reprogramming in CAR T cells through interactions with the mitochondrial fission protein dynamin-related protein 1, which is characterized by the concomitant down-regulation of aerobic glycolysis and oxidative phosphorylation with compensatory up-regulation of lipid metabolism; this metabolic signature markedly delays exhaustion upon repeated tumor antigen stimulation, enhances antisolid tumor efficacy, and notably does not confer the immunosuppressive functions of regulatory T cells [77,78]. In addition, logic gating technology can notably increase the specificity for senescent cells through the use of multiple antigen expression patterns, such as an AND gate design [79,80], in which CAR-engineered T lymphocytes require the concurrent engagement of 2 distinct antigenic targets to trigger functional responses, or an OR gate design [81], in which activation occurs upon recognition of either antigen. For example, prostate stem cell antigen and prostate-specific membrane antigen are common tumor-associated antigens in prostate cancer and are co-up-regulated in tumor tissues. The AND gate design notably narrows the off-target window through combinatorial recognition [82]. In addition, the urokinase-type plasminogen activator receptor (uPAR) + p16 dual-target AND gate strategy can be used to address the spatiotemporal heterogeneity of senescence markers. In summary, these key engineered strategies for CAR T cell updates lay a solid technical foundation for designing “smart” T cells that target senescent cells.

### From tumor antigens to senescence biomarkers: Target conversion

**Fig. 3.**
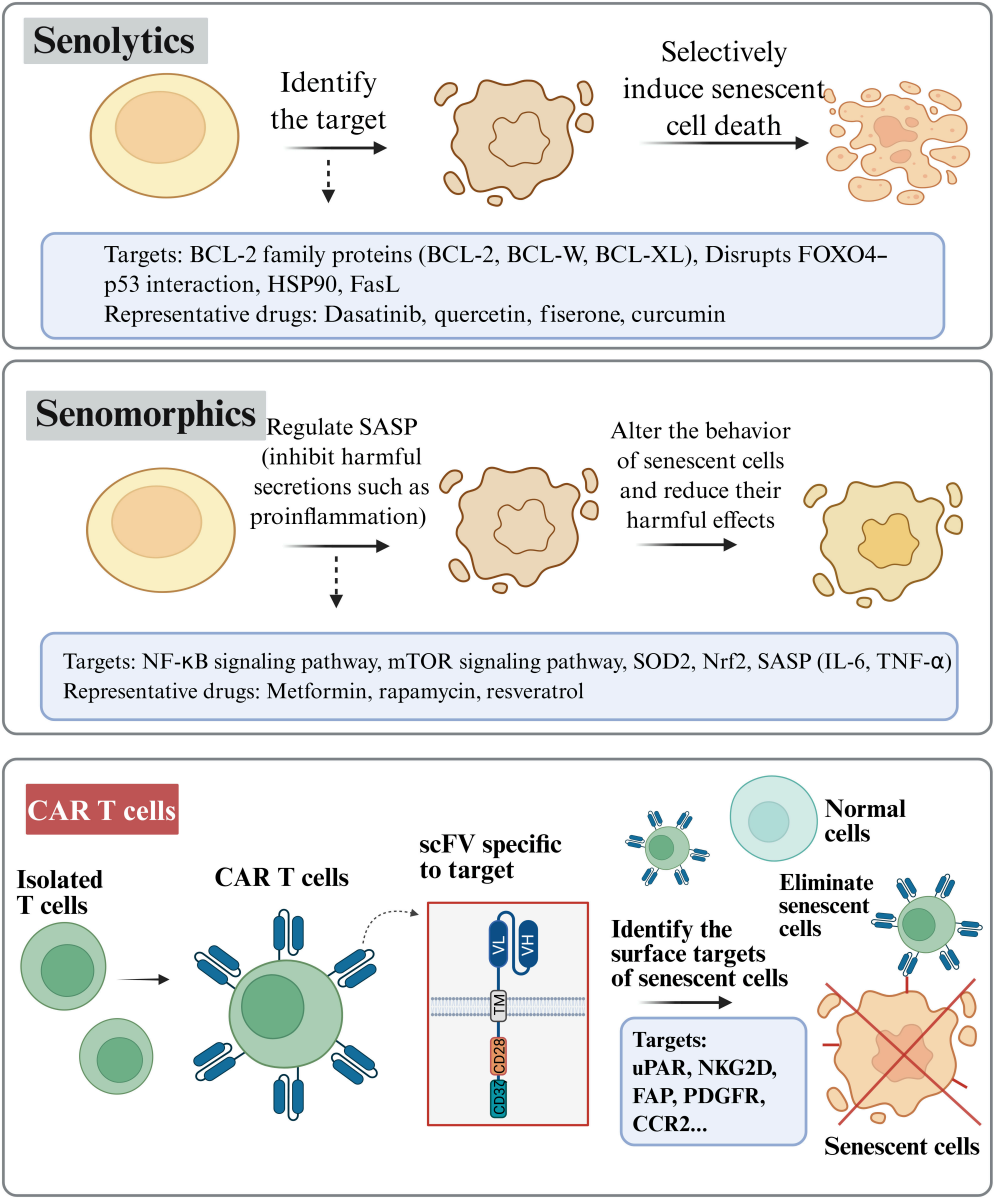
Mechanistic analysis of senolytic, senomorphic, and chimeric antigen receptor T cell (CAR T cell)-targeting senescent cells.FOXO4, forkhead box protein O4; HSP90, heat shock protein 90; FasL, Fas ligand; SOD, superoxide dismutase; Nrf2, nuclear factor erythroid 2-related factor 2; TM, tropomyosin; VL, immunoglobulin light chain variable domain; VH, immunoglobulin heavy-chain variable domain.

Senescent cells, as key drivers of age-related diseases, have become a hotspot in antiaging research because of their specific targeting and clearance ability. CAR-engineered T cell treatment falls within the immunotherapeutic modality class, which harnesses genetically reprogrammed patient T cells equipped with CAR modules for targeted neoplastic cell recognition and destruction (Fig. 3). Although this therapy was initially used primarily to treat cancer, in recent years, researchers have also explored its potential in age-related diseases, particularly certain immune-senescence-related conditions. The pivotal hurdle in translating CAR T cell therapy to senescence intervention lies in pinpointing robust, senescence-selective surface antigens with consistent expression profiles. This requires the integration of multiomics approaches, e.g., by comparing transcriptomic and proteomic data from young versus aged tissues or cells before and after induced senescence, for the systematic identification of molecules exhibiting selective enrichment on senescent cell membranes. Currently, multiple targets have shown promise. In Table 1, we list many senescence cell CAR T cell targets that have undergone preclinical validation.

**Table 1.** Summary of chimeric antigen receptor T cell (CAR T cell) candidate targets for senescence and fibrosis therapy along with their preclinical evidence

Target	Source/characteristics of target	Key preclinical evidence (experimental model and primary efficacy indicators)	Major risks and limitations	Applicable scenarios	Clinical translation prospects
Urokinase-type plasminogen activator receptor (uPAR) [87,88,152,153]	Multimodel RNA sequencing datasets, fish proteomics identification, GPI-anchored proteins on the surface of senescent cells	Constructed CAR T cells targeting uPAR, which can eliminate senescent hepatic stellate cells in liver fibrosis mouse models, alleviate fibrosis, and improve metabolic function with a single dose in aged/high-fat diet mouse models, demonstrating long-lasting effects	uPAR is also expressed in certain normal cells or cells during repair processes, necessitating rigorous evaluation of potential off-target toxicity.	Hepatic fibrosis, metabolic syndrome, physiological aging, tissue regeneration disorder	High, validated in multiple disease models; humanized CARs have been developed.
Natural killer group 2, member D (NKG2D) [92,94]	Stress-induced cell surface molecules recognized by the NKG2D receptor	Multiple in vitro and in vivo studies in mouse and nonhuman primate aging models have shown that NKG2D CAR can specifically eliminate stress-induced or senescent cells and significantly improve functional indicators.	Widely up-regulated in infections, inflammation, and cancer cells, posing specificity challenges; may be influenced by the human lymphocyte antigen-E (HLA-E)/NKG2A inhibitory axis	Aging related to viral infection, aging induced by DNA damage, aging induced by tumor treatment	Medium/high, requiring strict screening of eligible populations (e.g., those without active infections or tumors)
Fibroblast activation protein (FAP) [96,153]	RNA sequencing database; activated fibroblast/myofibroblast markers	FAP-targeted CAR T cell therapy in animal models effectively reduces tissue fibrosis, eliminates fibrogenic cells, and significantly improves organ function.	Expressed in fibroblasts during tissue repair phase; challenges in solid tumor delivery and immunosuppressive microenvironment	Cardiac fibrosis, hepatic fibrosis, idiopathic pulmonary fibrosis, rheumatoid arthritis/osteoarthritis (FAP + synovial cells)	Early clinical trials on local FAP CAR administration in humans for safety assessment have been initiated.
Platelet-derived growth factor receptor (PDGFRα/PDGFRβ)[154]	Platelet-derived growth factor receptor, a key factor in fibroblast activation	PDGFR signaling plays a pathogenic role in renal, cardiac, and hepatic fibrosis; preclinical reports on PDGFR-targeted CAR T cells are relatively limited, mainly involving theoretical exploration as a potential target and small-scale in vitro validation.	Expressed in pericytes and various normal stromal cells, posing high off-target risks	Hepatic fibrosis (targeting hematopoietic stem cell), tumor-associated matrix (targeting cancer-associated fibroblast); need to develop hematopoietic stem cell-specific CAR design	Medium/low, may require logic gating or localized administration to enhance safety
CCR2 [155,156]	Monocyte/macrophage-associated targets, indirectly regulating fibrosis	Inhibition models effectively alleviate fibrosis in the heart and other organs; CAR technology could be utilized to reprogram pro-fibrotic immune cells.	Immune cells are widely distributed.	Inflammatory aging, metabolic syndrome, tissue fibrosis (need to combine aging markers to improve specificity)	Medium/low, requiring highly controlled spatiotemporal and dosing strategies
Surface markers such as beta-2-microglobulin, CD36, and dipeptidyl peptidase-4 (DPP-4) [157]	Derived from multiomics analysis, serving as exploratory targets	Literature primarily involves omics and conceptual studies; some work is currently evaluating their feasibility as antibody–drug conjugates or other targeted strategies.	These molecules exhibit significant distribution differences across cell types and tissues.	Pulmonary fibrosis, skin aging (coexpression of FAP/DPP4), metabolic syndrome (combined DPP4 inhibition strategy)	Medium/low, requiring extensive validation to mitigate off-target toxicity risks

Emerging evidence from independent investigations indicates that the expression of uPAR, a glycosylphosphatidylinositol (GPI)-anchored protein, is up-regulated in various induced senescence models and is involved in the regulation of cell migration, invasion, and the SASP [83,84]. Asahina et al. [85] detected uPAR expression in the neuronal cytoplasm and vascular walls of subjects diagnosed with Alzheimer’s disease.

The multiomics screening pipeline is a complete process from discovery in transcriptomics to validation in proteomics, followed by confirmation at the single-cell level. Taking uPAR as an example, single-cell RNA sequencing revealed that the coexpression rate of uPAR and p16 exceeds 60%, and uPAR^+^ cells exhibit a typical senescence phenotype [86]. Amor and colleagues [87] conducted comparative analyses of transcriptomic datasets across 3 pivotal cellular senescence paradigms, combined them with the Human Protein Atlas and the Human Proteome Map, and identified uPAR as a specific candidate gene in critically aging tissues. With respect to functional validation, uPAR CAR T cells reduced the number of senescent cells by 87% and decreased collagen deposition by 52% in a liver fibrosis model and improved metabolic dysfunction and extended lifespan in naturally aged mice [88]. In in vitro models, flow cytometry revealed that the fluorescence intensity of uPAR in a senescence-induced lung adenocarcinoma cell group was significantly greater than that in a control group [89]. In in vivo experiments, coimmunostaining was used to detect uPAR [87] expression in senescent human hepatic stellate cells. In addition, uPAR-targeted CAR T cell antiaging experiments in mouse models demonstrated that CAR T cells significantly reduce the numbers of senescent tumor cells and hepatic fibrotic cells [87]. Studies have also shown that anti-uPAR CAR T cell therapy improved exercise capacity in physiologically aged mice and ameliorated metabolic dysfunction in aged and high-fat diet-fed mice, with long-term effects achieved through a single administration [88]. Other studies utilized anti-uPAR CAR T cells to alleviate hepatic fibrosis and rheumatoid arthritis and developed a humanized uPAR CAR encoded by cardiolipin-mimetic ionizable lipid 40 (PL40) mRNA, advancing its potential for treating aging-related inflammatory diseases in humans [90]. The high sequence and protein structural similarity of uPAR between mice and humans further support its clinical applicability.

Through transcriptomics and ImageStream imaging, Sagiv et al. [91] reported that the expression of the natural killer group 2, member D (NKG2D) ligands MICA and UL16 binding protein 2 was up-regulated twofold on the surface of senescent cells and that this up-regulation was positively correlated with the DDR. In vivo experiments confirmed that the NKG2D receptor–ligand interaction is essential for the ability of NK cells to clear senescent hepatic stellate cells and limit liver fibrosis; Nkg2d^-^/^-^ mice exhibited a 50% increase in the fibrotic area and a 45% accumulation of senescent cells [91]. As ligands for the NKG2D-activating receptor, MICA/B molecules are selectively displayed on stressed or malignant cellular surfaces. Experimental evidence from senescent mouse and primate systems has indicated that anti-NKG2DL CAR T cell therapy efficiently clears human cells that exhibit senescence hallmarks induced by diverse stressors—including oncogene activation, replication fork collapse, DNA double-strand breaks, and p16 pathway hyperactivation [92,93]. Deng et al. [94] engineered NKG2D CAR T cells that demonstrated potent cytolytic activity toward senescent murine embryonic fibroblasts and astrocytes but exhibited negligible cytotoxicity against their nonsenescent counterparts. In addition, platelet-derived growth factor receptor (PDGFR) molecules qualify as prospective targets for engineered CAR T cell engagement in various fibrosis and aging models (Table 1) [95]. In a mouse chronic kidney disease (CKD) model, adoptive transfer of PDGFRβ CAR T cells improved fibrosis-related pathological changes, including fibrosis in the kidney, myocardial interstitium, and perivascular areas, as reported by Zhao et al. [95]. In metabolic disease models, uPAR-CAR T cell therapy improved glucose tolerance and insulin sensitivity [87]. In fibrosis models, treatment with fibroblast activation protein (FAP) CAR T cells or PDGFRβ CAR T cells not only reduced collagen deposition but also improved physiological indicators such as the cardiac ejection fraction and renal function [95,96]. Engineered CAR T cells have also been explored for their immunomodulatory potential in neurodegenerative diseases. For example, CD4^+^ CAR T cells targeting amyloid-β were designed to reshape the immune microenvironment and promote clearance of extracellular amyloid plaques. This highlights the versatility of CAR T cell platforms beyond direct cytotoxicity against cellular targets [97]. These results preliminarily demonstrate that CAR T cell therapy can achieve tissue “repair” and functional recovery while “clearing” senescent cells. Not all senescence-associated targets are equally suitable for CAR T cell therapy, and target selection must be evaluated on the basis of expression profile, cellular localization, tissue distribution, and biological function in comparison with other targeted modalities, including T cell engagers, antibody–drug conjugates, and radiotherapy. Surface targets such as uPAR, NKG2DL, and dipeptidyl peptidase-4 (DPP4) are particularly preferable for CAR T cell platforms, as they are highly specific and consistently up-regulated in senescent cells across multiple tissues, mediate senescence-related immune recognition and SASP regulation, and are stably exposed on the cell membrane in the absence of internalization dependency.

Research on CAR T cell therapy for aging-related diseases is currently undergoing a strategic paradigm shift from broad-spectrum senescence markers to tissue-specific and senescence-stage-dependent targets. The emergence of a range of novel surface antigens provides a potential molecular basis for the precise elimination of senescent cells in pathological states and for intervening under degenerative conditions such as pulmonary fibrosis, OA, and mesenchymal stem cell (MSC) depletion. In pulmonary fibrosis research, LAMP1 has been shown to be a lysosomal-associated membrane protein whose expression is substantially up-regulated in both human and mouse senescent cells and is positively correlated with p16, p21, and the SASP [98]. Although the functional validation of LAMP1-targeting CAR T cells has not yet been reported, its surface localization and tissue distribution characteristics make it a theoretically feasible candidate target for pulmonary fibrosis CAR T cell therapy. In contrast, while adenine nucleotide translocase 1 is associated with a prosenescent phenotype in knockout mouse models and its knockdown can up-regulate the expression of senescence-associated genes in airway epithelial cells, its application prospects as a CAR-accessible surface target are limited because of its primary localization in the inner mitochondrial membrane [99]. In MSC senescence research, CD264 has been confirmed to be a surface marker that is up-regulated during the middle stage of MSC senescence, and Kremen1 serves as a newly identified MSC-specific senescence surface antigen; both of these findings demonstrate its theoretical feasibility as an immunotherapeutic target at the cellular level [100]. Moreover, although lysine acetyltransferase 8 can inhibit p21 and p16 expression through epigenetic regulation and delay human umbilical cord MSC senescence, its nuclear localization renders it more amenable to gene editing intervention than to CAR T cell recognition strategies [101]. In the field of OA, DPP4 has been validated as a functional surface marker of senescent chondrocytes; DPP4-positive cells exhibit a typical senescent phenotype and SASP characteristics, and antibody-mediated clearance of DPP4-positive cells significantly improves the pathological phenotype in OA animal models, providing a conceptual validation basis for CAR T cell translation targeting this molecule [102]. disialoganglioside 3, a ganglioside surface molecule, is up-regulated in the joints of patients with OA, and disialoganglioside 3-positive chondrocytes and synovial cells also display a high senescent burden (Table S1) [103]. Although still in the fundamental research stage, its glycolipid molecular attributes offer a theoretical possibility for CAR T cell targeting. In summary, the commonality among the aforementioned targets lies in their tissue-specific expression patterns and senescence-stage-dependent up-regulation characteristics, offering a theoretical advantage for precisely eliminating senescent cells and mitigating the off-target toxicity associated with broad-spectrum clearance. However, most current research remains confined to preliminary validation stages in in vitro cellular models or at the animal level and has not yet progressed to CAR-T-cell-specific preclinical functional assessment and safety validation phases. Critical issues, including target immunogenicity, antigen escape risk, and engineering adaptation strategies, still warrant rigorous systematic investigation.

## From Cellular Clearance to Tissue Repair: Reversing Fibrosis and Multiorgan Dysfunction

Fibrosis represents a dysregulated tissue repair mechanism triggered by persistent insult, hallmarked by pathological accumulation of extracellular matrix components, and constitutes a central pathological outcome across diverse aging-associated disorders, such as CKD [104,105], cardiac fibrosis and heart failure [106], and pulmonary fibrosis [107]. Growing evidence suggests that senescent cells are key drivers of progressive fibrosis [108]. In the fibrotic process, fibroblasts are key effector cells that promote fibroblast activation through signaling pathways such as the Wnt/β-catenin [109], TGF-β [110,111], and PDGF [112] pathways, thereby increasing extracellular matrix deposition and exacerbating tissue fibrosis. Consequently, therapeutic strategies aimed at eliminating senescent cells or their downstream myofibroblast derivatives have garnered substantial interest as a means to reverse fibrotic pathology, with CAR T cell immunotherapy offering distinct advantages in this emerging domain. FAP is a 97-kD type II transmembrane serine protease that is typically expressed in fibroblasts, myofibroblasts, and certain cancer-associated fibroblasts [113,114] and serves as a representative marker of fibroblasts. Multiple studies have demonstrated that CAR T cells expressing anti-FAP antibodies can target FAP-expressing myofibroblasts, substantially alleviating cardiac fibrosis in mice and restoring postinjury function [96,115]. Furthermore, FAP-targeted CAR T cells reduce fibroblast numbers in pulmonary fibrosis mouse models, mitigate the degree of lung fibrosis, and thereby improve pulmonary function [113]. This “repair” effect may stem from multiple mechanisms: The clearance of pathological cells directly removes mechanical and biochemical barriers; the elimination of the SASP alleviates local chronic inflammation; and the improved microenvironment may facilitate the reparative functions of endogenous progenitor or stem cells. CAR T cell therapy thus represents a transition from purely “cytotoxic” to “tissue-repair” immunotherapy, with the goal of eradicating injurious cells while restoring normal organ morphology and operation.

## CAR T Cell Therapy Targeting Senescent Cells: Comparison with Other Senotherapies

In addition to CAR T cell therapy, several treatments targeting age-related diseases, such as senolytics, senomorphics, and stem cell therapy, have shown potential, many of which have entered multiple clinical trial stages. Senolytic drugs treat age-related diseases by targeting and eliminating senescent cells, thereby reducing inflammation, oxidative stress, and other issues (Fig. 3) [13]. Currently, most senolytic drugs, such as dasatinib and quercetin and fisetin and quercetin, remain in clinical trials, with some having progressed to phase 1 or 2 trials [116,117]. Patients with diabetic kidney disease administered oral dasatinib and quercetin exhibited substantial reductions in multiple senescence-associated parameters, such as the adipose tissue senescent cell load, inflammation, fibrosis, and circulating SASP mediators, which were evident within 11 d of treatment cessation [118,119]. First-generation oral antiaging drugs typically target the antiapoptotic pathways of senescent cells and are mostly natural products with known safety profiles. In addition, research has utilized new methods, such as machine learning, for screening senolytic targets and drugs [120]. Although most senolytic drugs are still in clinical trials and lack large-scale clinical data support, challenges remain in precisely selecting senescent cells for clearance and determining safe and effective dosing regimens. Senomorphic drugs do not directly clear senescent cells but instead regulate their function (by inhibiting SASP production) to maintain senescent cells in a “nonharmful” state, with the goal of reducing the release of harmful factors by senescent cells (Fig. 3) [15,121,122]. The encouraging preclinical performance of senomorphic interventions stands in contrast to persistent translational obstacles: inadequate target discrimination, safety concerns, and unresolved clinical implementation pathways. Since they cannot completely eliminate senescent cells, senomorphics require long-term administration to sustain the suppression of the SASP [15]. Stem cell therapy involves injecting or activating stem cells to repair aged or damaged tissues, thereby improving aging symptoms and function [123,124].

We systematically compared these common antiaging interventions in terms of their mechanisms of action, durability of efficacy, and convenience (Table 2). Compared with CAR T cell therapy, antiaging drugs that have entered clinical trials generally suffer from short-lived efficacy and an inability to precisely identify and target senescent cells at the cellular level. CAR-engineered T lymphocytes, which represent a paradigm shift in immunotherapeutic modalities, exhibit substantial promise for eliminating senescent cells because of their exquisite target specificity and durable therapeutic activity.

**Table 2.** Comparison of the effects of chimeric antigen receptor T cell (CAR T cell) therapy and traditional biologics on age-related diseases

Characteristics	CAR T cell therapy	Senolytics	Senomorphics	Stem cell therapy
Mechanism of action	Utilizing gene-editing technology, T cells can precisely recognize and effectively eliminate tumor cells or senescent cells.	Targets survival signaling pathways such as antiapoptotic proteins (e.g., B cell lymphoma-2 and BCL-XL) highly expressed in senescent cells, selectively clearing these senescent cells.	Modulates the behavior of senescent cells to alleviate their harmful effects	Replaces or repairs aged tissues, promoting tissue regeneration
Targeting specificity	High specificity achieved through scFv and logic gating.	Medium/low, relying on differences in drug-sensitive pathways between senescent and nonsenescent cells, with potential off-target risks	Low, typically targeting widely expressed signaling pathways, affecting normal cell function	Low, mostly paracrine or differentiating into local cell types, nonspecifically targeting senescent cells
Durability of efficacy	Potentially durable, with a single infusion possibly providing long-term effects (dependent on cell survival in vivo)	Transient, requiring regular intermittent dosing, as senescent cells may reaccumulate	Dependent on continuous administration; SASP may resume after discontinuation.	Depends on stem cell survival, homing, and integration, potentially requiring repeated infusions
Convenience	Individualized preparation, complex and time-consuming process, costly	Oral or injectable administration, convenient, relatively low cost	Typically, small molecules or biologics, convenient administration	Complex and expensive cell collection, expansion, and quality control processes
Current main application stage	Mature in cancer treatment; senescence therapy is in preclinical and early exploratory stages.	Multiple phase 1/2 clinical trials targeting specific age-related diseases (e.g., pulmonary fibrosis, diabetic nephropathy)	Most are in the preclinical research stage, with a few entering early clinical exploration.	Clinical studies on some degenerative diseases show inconsistent efficacy.
Side effects/risks	Cytokine release syndrome, neurotoxicity, on-target/off-tumor toxicity, long-term safety unknown	Off-target cytotoxicity, limited long-term medication safety data, potential impact on tissue repair	Interference with normal physiological inflammatory responses, unknown consequences of long-term suppression of specific pathways	Immune rejection, tumorigenic risk, abnormal differentiation, embolism, etc.

## Challenges and Optimization: Paving the Way for Clinical Applications

### Challenges in target specificity and safety

Senescent cells are not completely pathological in vivo; they also play important roles in tissue repair, embryonic development, and immune regulation. To avoid “in vivo toxicity”, any CAR T cell therapy targeting senescence markers needs to identify cells with “normal but low-level expression of senescence markers”. For example, uPAR is also expressed in some normal or repairing cells (potential “off-target toxicity"); NKG2D ligands may be up-regulated in infected, inflammatory, or transformed cells; and FAP is expressed in certain fibroblast subpopulations during healing or regeneration processes (Table 1). In addition, senescence-associated β-galactosidase (SA-β-Gal), which is highly expressed in senescent cells, is present in cells with high lysosomal activity [125]. Furthermore, senescent cells play different roles under various physiological states and exhibit physiological functions in tissue repair, embryonic development, liver injury repair, and skin regeneration [126].

Therefore, it is necessary to conduct more precise and specific target screening and validation of senescent cells to reduce risks. By integrating transcriptomics, surface proteomics, spatial transcriptomics, and the SASP, we aimed to identify candidate molecules that are stably and significantly enriched on the surface proteins of senescent cells but are minimally expressed or absent in critical normal tissues. For example, studies have collected samples from healthy controls and patients with Parkinson’s disease to construct single-cell transcriptomic and epigenomic maps of the substantia nigra. Multiomics analyses have revealed that cell-type-specific transcriptional regulation is associated with Parkinson’s disease [127]. Since senescence phenotypes vary depending on the inducing factors and tissues, it is essential to identify “highly common” markers in the target pathological context, covering a broad range of age-related disease targets. Next, target prioritization and functional and safety validation (including multisource cell or tissue validation and in vitro functional assessments) should be performed, along with the preparation of CAR engineering and safety mitigation strategies. To address the on-target off-tumor toxicity of uPAR targeting, we propose the implementation of synNotch-based AND gate circuits that require dual antigen recognition. The synNotch receptor recognizes uPAR and induces CAR expression only upon binding, with a 24-h delay ensuring spatially confined activation [128,129]. SynNotch receptors recognize uPAR on the cell surface and activate downstream transcriptional cascades, inducing surface reporter proteins such as truncated epidermal growth factor receptor or engineered CD19 driven by the p16 promoter. The AND gate logic is fully functional only when cells simultaneously express uPAR to trigger synNotch signaling and exhibit high p16 transcriptional activity to drive reporter protein expression. This strategy converts intracellular p16 status into detectable surface antigens yet requires the introduction of additional synthetic genetic circuits. Triple AND gates (uPAR, p16, and additional markers) can further increase specificity for high-risk applications.

### Challenges of the aged immune environment and cell preparation

**Fig. 4.**
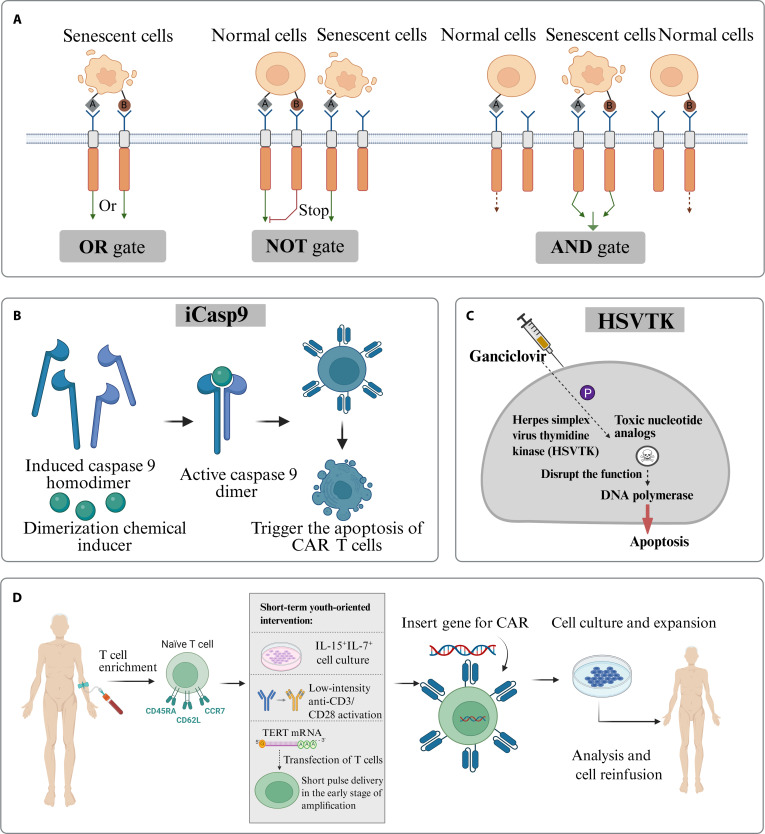
Schematic diagram of chimeric antigen receptor T cell (CAR T cell) strategies for targeting senescent cells. (A) Logic gating: OR gate, NOT gate, and AND gate. (B and C) Typical structural designs of the regulatable switch inducible caspase 9 (iCasp9) and the irreversible suicide system herpes simplex virus thymidine kinase (HSVTK). (D) CAR T cell manufacturing process using aged donors.

Elderly patients have a fragile baseline immune environment and commonly experience immune homeostasis imbalance and chronic inflammation [130]. Normal cell damage can easily trigger severe fibrosis or organ failure. Therefore, when CAR T cell therapies for age-related diseases are being developed, controllable switches (such as drug-inducible elimination mechanisms, light control, and ultrasound control) and suicide genes are absolutely essential in CAR T cell applications that target senescent cells. One study developed a drug-regulated system called signal-neutralizing inhibitory protease, in which snipcar can remotely regulate CAR activity through drug withdrawal, serving as an effective switch [131]. Suicide genes such as iCasp9 [132,133] and herpes simplex virus thymidine kinase (HSVTK) [134] can rapidly terminate the function of the entire CAR T cell population during cytokine storms or local tissue damage, preventing irreversible harm (Fig. 4).

With advancing age, significant T cell remodeling occurs in the population. Owing to age-related thymic degeneration, the number of naïve cells decreases, whereas cells with memory/effector or senescent-like phenotypes gradually accumulate, accompanied by decreases in cell proliferation and secretory functions. In addition, the progressive shortening of telomeres not only limits the replicative lifespan of cells but also accelerates premature cellular senescence. These changes impair the quality of CAR T cells prepared from elderly donors [135–137]. By screening for “young/high-quality” subpopulations in aged peripheral blood, such as classical naïve cells (CD45RA^+^ CCR7^+^ CD62L^+^), cell quality can be effectively enhanced. We recommend the following: (a) IL-7-based culture using efineptakin alfa, which increases the proportion of CD4^+^ CAR T cells by 66% and that of T stem cell memory cells by 133% while reducing the expression of exhaustion markers by 40% [138]; (b) mTOR inhibition with low-dose rapamycin (1 to 5 mg/week) to promote metabolic reprogramming toward oxidative phosphorylation and memory differentiation [139]; and (c) telomerase reverse transcriptase (TERT) mRNA electroporation to transiently extend telomeres by 0.9 kb and increase the proliferative potential through 43 population doublings without increasing the risk of immortalization. The combination of these strategies may synergistically rejuvenate aged T cells for CAR T cell manufacturing (Fig. 4D) [140].

Aging tissues typically exhibit various immunosuppressive characteristics, such as the SASP, up-regulation of immune checkpoint genes (programmed cell death ligand 1, adenosine generation pathways, etc.) [141], decreased costimulatory signals, and metabolic and microenvironmental stresses (hypoxia, high lactate levels, low nutrient levels, and increased reactive oxygen species levels) [142], which directly impact the efficacy of CAR T cells [143]. To enhance cellular efficacy, cytokine receptors such as IL-7 receptor (IL-7R) and IL-15R or receptors that enhance IL-2 signaling can be expressed on CAR T cells to improve their survival and maintenance of memory-like phenotypes [144]. Alternatively, localized secretion or “armoring” factors (IL-15, IL-12, etc.) can be used to remodel the local immune microenvironment, thereby enhancing cellular function [145].

Beyond individual aging-associated immune environments, CAR T cell therapy itself can induce treatment-related inflammation, thereby reshaping the local immune microenvironment. In studies of human multiple myeloma, anti-B cell maturation antigen CAR T cell therapy was shown to remodel the endogenous T cell repertoire, with the emergence of a novel transitional CD8^+^ T cell population whose enrichment correlated with poor prognosis [146]. Further investigations revealed that post-CAR-T-cell enrichment of PD-1^+^ endogenous T cells within the bone marrow microenvironment was associated with a lack of durable response, while the T cell immunoglobulin and mucin domain-containing protein 3 (TIM3)/galectin-9 interaction was identified as a potential intervention target [146]. In elderly individuals, increased bone marrow adiposity, hematopoietic stem cell exhaustion, and mesenchymal stromal cell senescence collectively constitute a microenvironment with diminished immune-supportive capacity, potentially restricting CAR T cell homing, expansion, and long-term persistence. On the basis of these findings, targeting the TIM3/galectin-9 axis or improving the bone marrow niche—such as through senolytic-mediated clearance of senescent stromal cells—may represent novel strategies to increase CAR T cell durability in aged hosts.

### Limitations of preclinical models and translational challenges

Preclinical models of aging also have limitations. Current preclinical models include accelerated aging mice and naturally aged mice [147]. Compared with real aged tissues, the accelerated aging process in mice is nonnatural, leading to differences in pathology and the immune microenvironment, as well as variations in immune system development, which may affect the predictive value of CAR T cell therapy [148]. Moreover, naturally aged mouse models are limited in terms of the types of pathologies they develop, making it difficult to fully replicate complex multisystem geriatric diseases. Finally, when genetically engineered cell therapies are applied to nonfatal age-related diseases, safety and controllability must take precedence over efficacy, which is often reflected in chronic cumulative improvements in quality of life or functional outcomes. Therefore, safety, controllability, long-term effects, and societal ethical considerations become the core of regulatory approval rather than efficacy alone. Current mouse models inadequately replicate human aging. We advocate the integration of NOD scid gamma–human stem cell factor, granulocyte-macrophage colony-stimulating factor, and interleukin-3 (NSG-SGM3) humanized mice with patient-derived senescent cell xenografts for efficacy validation and nonhuman primate aging models for long-term safety assessment prior to clinical translation.

## Future Prospects: Toward Precision, Controllability, and Systematic Antiaging Immunotherapy

In the future, mRNA–lipid nanoparticle technology is expected to be used to directly generate transient antiaging CAR T cells in vivo, transforming CAR T cells from a traditional “cell therapy” to a drug-like form. This strategy will substantially increase treatment accessibility and flexibility, eliminating the need for in vitro expansion and complex cell preparation processes while providing short-term and controllable immune intervention. Artificial intelligence (AI) and machine learning technologies can be applied to predict optimal antigen combinations, CAR structures, signaling logic circuits, and safety switch designs, thereby achieving personalized and precise antiaging CAR T cell therapy. AI models—particularly graph neural networks and Transformer architectures—enable deep interrogation of large-scale single-cell transcriptomic sequencing datasets, facilitating precise identification of senescent-cell-specific surface antigen signatures across diverse tissue microenvironments, including the lung, cartilage, and bone marrow, while systematically evaluating their theoretical feasibility and therapeutic potential as CAR T cells. Specifically, machine learning algorithms have been successfully deployed to mine single-cell transcriptomic data from CD8^+^ T cells, effectively revealing age-associated transcriptional signatures, with further extension toward predicting the differentiation stage and clinical efficacy outcomes of adoptively transferred CAR T cells [149]. AI-assisted design not only accelerates the screening and optimization of candidate molecules but also reduces experimental costs and improves efficacy and safety. AI harnesses machine learning and deep learning algorithms to uncover hidden patterns, predict the onset of senescence, and enable precise classification of aging cells from imaging or omics data [150]. AI-driven design platforms, exemplified by CAR-Toner, analyze massive protein sequence datasets—encompassing tens of thousands of entries—to rapidly compute positive charge patch scores, thereby providing intelligent guidance for screening antigen-binding domains with low immunogenicity and high structural stability [151].

A critical barrier to the clinical translation of senolytic CAR T cells in aging populations lies in the intrinsic defects of aged T cells, including epigenetic dysregulation (e.g., reduced H3 lysine 4 trimethylation [H3K4me3] and increased H3K9me3 levels), metabolic remodeling (mitochondrial dysfunction and impaired autophagy), and a predisposition to exhaustion (up-regulation of PD-1, TIM3, lymphocyte-activation gene 3, and thymocyte selection-associated high mobility group box protein expression). These changes collectively impair CAR T cell expansion, persistence, and effector function. To actively reshape the proinflammatory senescent microenvironment, advanced CAR technologies offer a promising solution. For instance, CAR T cells can be engineered to secrete neutralizing nanobodies or scFvs against key SASP components (IL-6, IL-1β, tumor necrosis factor-α [TNF-α], and C–C motif chemokine ligand 2 ). Preclinical evidence in fibrosis models has indicated that such advanced CARs reduce collagen deposition by up to 45% and lower serum SASP factors by 70% to 80% while improving aged T cell proliferation and mitochondrial function. Potential risks (systemic immunosuppression, vector capacity, and immunogenicity) can be mitigated by conditional secretion, AND gate targeting, humanized sequences, and suicide switches. These strategies collectively address both the senescent target and the hostile aged immune microenvironment.

Although traditional senolytics—including dasatinib–quercetin combinations and fisetin—achieve systemic senescent cell clearance, they are limited by transient pharmacodynamics and limited tissue selectivity. Conversely, CAR T cells provide durable, antigen-specific immune surveillance but suffer from delayed onset kinetics. The sequential combination of these modalities enables therapeutic temporal synergy: An initial senolytic phase rapidly reduces the systemic senescent burden and attenuates the SASP-driven inflammatory milieu, followed by CAR T cell therapy to eradicate residual senescent populations and establish long-term immune memory, thereby achieving sustained clearance efficacy. Although CAR T cells targeting senescence remain preclinical, NKG2D CAR T cells have shown robust senolytic activity in aged nonhuman primates with favorable safety profiles, and ongoing oncology trials (e.g., NCT05653206) provide supporting safety data. In addition to oral senolytics, recent phase 1 trials of the use of dasatinib plus quercetin in the treatment of Alzheimer’s disease have demonstrated a reduction in central nervous system penetration and SASP. Collectively, these advances support the feasibility of the near-term clinical translation of CAR T cells for the treatment of senescence-associated diseases.

The application of CAR T cell technology is driving the transition of medicine from “treating single diseases” to targeting common aging processes. By precisely regulating the immune system and microenvironment, CAR T cells have the potential to delay multisystem functional decline and achieve the ultimate goal of healthy aging. From a translational perspective, future research should also focus on addressing clinical translation pathways. Age-related diseases with substantially accumulation of senescent cells and high unmet clinical needs, such as idiopathic pulmonary fibrosis, may serve as ideal initial indications. When early clinical trials are designed, endpoint settings should go beyond mere aging-related molecules or circulating biomarkers and encompass clinically meaningful outcomes, including functional improvements, organ-specific manifestations, and patient-reported quality of life. This multidimensional endpoint framework is crucial for accurately capturing treatment effects and advancing regulatory evaluations. This cutting-edge field urgently requires interdisciplinary collaboration, deeply integrating aging biology, immunology, and genetic engineering to develop systematic antiaging therapeutic strategies.

## Supplementary Materials

Supplementary 1 Table S1 Reference [158]

## References

[1] Martin LG. Population aging policies in East Asia and the United States. Science. 1991;251(4993):527–531. 1990428 10.1126/science.1990428

[2] Yuan J, Zhang S, Han D, Dong X. Agrin at the crossroads of aging: A pleiotropic regulator in age-related diseases. Pharmacol Res. 2026;225: Article 108131. 41654149 10.1016/j.phrs.2026.108131

[3] Sanfeliu-Redondo D, Gibert-Ramos A, Gracia-Sancho J. Cell senescence in liver diseases: Pathological mechanism and theranostic opportunity. Nat Rev Gastroenterol Hepatol. 2024;21(7):477–492. 38485755 10.1038/s41575-024-00913-4

[4] Wang B, Han J, Elisseeff JH, Demaria M. The senescence-associated secretory phenotype and its physiological and pathological implications. Nat Rev Mol Cell Biol. 2024;25(12):958–978. 38654098 10.1038/s41580-024-00727-x

[5] Borodkina AV, Deryabin PI, Giukova AA, Nikolsky NN. “Social life” of senescent cells: What is SASP and why study it? Acta Nat. 2018;10(1):4–14. PMC5916729 29713514

[6] Lucas V, Cavadas C, Aveleira CA. Cellular senescence: From mechanisms to current biomarkers and senotherapies. Pharmacol Rev. 2023;75(4):675–713. 36732079 10.1124/pharmrev.122.000622

[7] Di Micco R, Krizhanovsky V, Baker D, d’Adda di Fagagna F. Cellular senescence in ageing: From mechanisms to therapeutic opportunities. Nat Rev Mol Cell Biol. 2021;22(2):75–95. 33328614 10.1038/s41580-020-00314-w PMC8344376

[8] Birch J, Gil J. Senescence and the SASP: Many therapeutic avenues. Genes Dev. 2020;34(23-24):1565–1576. 33262144 10.1101/gad.343129.120 PMC7706700

[9] Wei W, Ji S. Cellular senescence: Molecular mechanisms and pathogenicity. J Cell Physiol. 2018;233(12):9121–9135. 30078211 10.1002/jcp.26956

[10] Campisi J, Kapahi P, Lithgow GJ, Melov S, Newman JC, Verdin E. From discoveries in ageing research to therapeutics for healthy ageing. Nature. 2019;571(7764):183–192. 31292558 10.1038/s41586-019-1365-2 PMC7205183

[11] Gasek NS, Kuchel GA, Kirkland JL, Xu M. Strategies for targeting senescent cells in human disease. Nat Aging. 2021;1(10):870–879. 34841261 10.1038/s43587-021-00121-8 PMC8612694

[12] Suda M, Paul KH, Tripathi U, Minamino T, Tchkonia T, Kirkland JL. Targeting cell senescence and senolytics: Novel interventions for age-related endocrine dysfunction. Endocr Rev. 2024;45(5):655–675. 38500373 10.1210/endrev/bnae010 PMC11405506

[13] Chaib S, Tchkonia T, Kirkland JL. Cellular senescence and senolytics: The path to the clinic. Nat Med. 2022;28(8):1556–1568. 35953721 10.1038/s41591-022-01923-y PMC9599677

[14] Wang X, Guo C, Shao J, Zou X, Xing S, Xu CL, Zhao Q, Wu Y, Sun C, Chen Y, et al. Small molecule-drug conjugates: An emerging drug design strategy for targeted therapeutics. J Med Chem. 2025;68(23):24759–24784. 41273282 10.1021/acs.jmedchem.5c01861

[15] Alum EU, Izah SC, Uti DE, Ugwu OP, Betiang PA, Basajja M, Ejemot-Nwadiaro RI. Targeting cellular senescence for healthy aging: Advances in senolytics and senomorphics. Drug Des Devel Ther. 2025;19:8489–8522. 10.2147/DDDT.S543211 PMC12456441 40994903

[16] Gu R, Shen J, Zhang J, Mao J, Ye Q. Revolutionizing autoimmune kidney disease treatment with chimeric antigen receptor-T cell therapy. Research. 2025;8:0712. 40405911 10.34133/research.0712 PMC12095914

[17] Yu B, Xu J, Cui Y. From technological iteration to clinical breakthrough: Advances of CAR-T cell therapy in autoimmune diseases. Ann Med. 2026;58(1):2627057. 41657283 10.1080/07853890.2026.2627057 PMC12922707

[18] Faramand RG, Xie Z, Jain MD. Long-term survivorship after chimeric antigen receptor T-cell therapy for hematologic malignancies. J Clin Oncol. 2026;44(8):698–708. 41671523 10.1200/JCO-25-02087

[19] Davis KL, Yao CC, Zimmerman JAO, Rau RE. Immunotherapy in B-cell acute lymphoblastic leukemia. J Natl Compr Cancer Netw. 2025;23(12): Article e257067. 10.6004/jnccn.2025.7067 41671463

[20] Bui TA, Mei H, Sang R, Ortega DG, Deng W. Advancements and challenges in developing in vivo CAR T cell therapies for cancer treatment. EBioMedicine. 2024;106: Article 105266. 39094262 10.1016/j.ebiom.2024.105266 PMC11345408

[21] Kong Y, Li J, Zhao X, Wu Y, Chen L. CAR-T cell therapy: Developments, challenges and expanded applications from cancer to autoimmunity. Front Immunol. 2024;15:1519671. 39850899 10.3389/fimmu.2024.1519671 PMC11754230

[22] Zhao Z, Chen Y, Francisco NM, Zhang Y, Wu M. The application of CAR-T cell therapy in hematological malignancies: Advantages and challenges. Acta Pharm Sin B. 2018;8(4):539–551. 30109179 10.1016/j.apsb.2018.03.001 PMC6090008

[23] Mougiakakos D, Krönke G, Völkl S, Kretschmann S, Aigner M, Kharboutli S, Böltz S, Manger B, Mackensen A, Schett G. CD19-targeted CAR T cells in refractory systemic lupus erythematosus. N Engl J Med. 2021;385(6):567–569. 34347960 10.1056/NEJMc2107725

[24] Mackensen A, Müller F, Mougiakakos D, Böltz S, Wilhelm A, Aigner M, Völkl S, Simon D, Kleyer A, Munoz L, et al. Anti-CD19 CAR T cell therapy for refractory systemic lupus erythematosus. Nat Med. 2022;28(10):2124–2132. 36109639 10.1038/s41591-022-02017-5

[25] Lai M, Shao W, Mao J, Ye Q. Revolution in cell therapy: In vivo chimeric-antigen-receptor-T-cell therapy breakthroughs and promises for the future. Research. 2025;8: Article 0917. 41079670 10.34133/research.0917 PMC12509061

[26] Zheng X, Zhao Y, Liu Z. Neoantigen identification and TCR-T therapy development for solid tumors: Current advances and future perspectives. J Natl Cancer Cent. 2025;5(5):429–440. 41111930 10.1016/j.jncc.2025.07.001 PMC12529609

[27] Zhu S. CAR-T in cancer therapeutics and updates. J Natl Cancer Cent. 2024;4(3):189–194. 39281717 10.1016/j.jncc.2024.01.001 PMC11402450

[28] Singh SB, Bhandari S, Siwakoti S, Kumar M, Singh R, Bhusal S, . Sharma K, Bhandari S, Khanal K. PET/CT in the evaluation of CAR-T cell immunotherapy in hematological malignancies. Mol Imaging. 2024;23:15353508241257924. 38952399 10.1177/15353508241257924 PMC11208886

[29] Pereira B, Correia FP, Alves IA, Costa M, Gameiro M, Martins AP, . Saraiva JA. Epigenetic reprogramming as a key to reverse ageing and increase longevity. Ageing Res Rev. 2024;95: Article 102204. 38272265 10.1016/j.arr.2024.102204

[30] Cipriano A, Moqri M, Maybury-Lewis SY, Rogers-Hammond R, de Jong TA, Parker A, Rasouli S, Schöler HR, Sinclair DA, Sebastiano V. Mechanisms, pathways and strategies for rejuvenation through epigenetic reprogramming. Nature aging. 2024;4(1):14–26. 38102454 10.1038/s43587-023-00539-2 PMC11058000

[31] Bloom SI, Karlseder J. Telomeres at the nexus of aging, tumor suppression, and inflammation: Toward an understanding beyond senescence. Genes Dev. 2025;39(15-16):920–922. 40645667 10.1101/gad.353122.125 PMC12315855

[32] López-Otín C, Pietrocola F, Roiz-Valle D, Galluzzi L, Kroemer G. Meta-hallmarks of aging and cancer. Cell Metab. 2023;35(1):12–35. 36599298 10.1016/j.cmet.2022.11.001

[33] Khemka S, Reddy A, Garcia RI, Jacobs M, Reddy RP, Roghani AK, Pattoor V, Basu T, Sehar U, Reddy PH. Role of diet and exercise in aging, Alzheimer’s disease, and other chronic diseases. Ageing Res Rev. 2023;91: Article 102091. 37832608 10.1016/j.arr.2023.102091 PMC10842571

[34] Andronie-Cioara FL, Ardelean AI, Nistor-Cseppento CD, Jurcau A, Jurcau MC, Pascalau N, Marcu F. Molecular mechanisms of neuroinflammation in aging and Alzheimer’s disease progression. Int J Mol Sci. 2023;24(3):1869. 36768235 10.3390/ijms24031869 PMC9915182

[35] Hong S, Baek SH, Lai MKP, Arumugam TV, Jo DG. Aging-associated sensory decline and Alzheimer’s disease. Mol Neurodegener. 2024;19(1):93. 39633396 10.1186/s13024-024-00776-y PMC11616278

[36] Tang L, Wang P, Sheng S, Yang H, Jiang Y, Jing Y, Liu H, Su J. Extracellular vesicles in bone aging: Therapeutic strategies and applications. Extracell Vesicles Circ Nucleic Acids. 2025;6(4):687–723. 10.20517/evcna.2025.58 PMC12809412 41551592

[37] Sølling AS, Langdahl BL, Cosman F. Recent advances in osteoporosis therapeutics. Annu Rev Med. 2026;77(1):433–448. 41252575 10.1146/annurev-med-050124-040555

[38] Shushpanova TV, Bokhan NA, Smirnova IN, Gameeva EV, Stepanova AM, Novozheeva TP, Kazennykh TV, Shushpanova OV, Safronov SM, Boev RG, et al. Vascular dysfunction: Processes due to aging, approaches to restorative therapy and prevention (part 1). Adv Gerontol. 2025;38(4):612–623. 41477787

[39] Herman AB, Candia J, Wilson DM 3rd, Donega S, Picard M, Ferrucci L. Molecular damage associated with ageing drives inflammation in cardiovascular disease. Nat Rev Cardiol. 2026;1–4. 41571994 10.1038/s41569-026-01253-3 PMC12951825

[40] Zhong D, Fernández-García B, Gokulnath P, Lin K, Li G, Kroemer G, López-Otín C, Xiao J. Exercise and the hallmarks of cardiovascular aging. Ageing Res Rev. 2026;115: Article 103030. 41580156 10.1016/j.arr.2026.103030

[41] Yu Y, Sun Y, Zhao T. The roles of glycerophospholipids in the aging retina and age-related macular degeneration. Graefes Arch Clin Exp. 2025;264:309–323. 10.1007/s00417-025-07011-4 41231253

[42] Cvekl A, Vijg J. Aging of the eye: Lessons from cataracts and age-related macular degeneration. Ageing Res Rev. 2024;99: Article 102407. 38977082 10.1016/j.arr.2024.102407 PMC11288402

[43] Zhang M, Serna-Salas S, Damba T, Borghesan M, Demaria M, Moshage H. Hepatic stellate cell senescence in liver fibrosis: Characteristics, mechanisms and perspectives. Mech Ageing Dev. 2021;199: Article 111572. 34536446 10.1016/j.mad.2021.111572

[44] Liu Z, Liang Q, Ren Y, Guo C, Ge X, Wang L, Cheng Q, Luo P, Zhang Y, Han X. Immunosenescence: Molecular mechanisms and diseases. Signal Transduct Target Ther. 2023;8(1):200. 37179335 10.1038/s41392-023-01451-2 PMC10182360

[45] Behmoaras J, Gil J. Similarities and interplay between senescent cells and macrophages. J Cell Biol. 2021;220(2): Article e202010162. 33355620 10.1083/jcb.202010162 PMC7769159

[46] Mohamad Kamal NS, Safuan S, Shamsuddin S, Foroozandeh P. Aging of the cells: Insight into cellular senescence and detection methods. Eur J Cell Biol. 2020;99(6): Article 151108. 32800277 10.1016/j.ejcb.2020.151108

[47] Hernandez-Segura A, Nehme J, Demaria M. Hallmarks of cellular senescence. Trends Cell Biol. 2018;28(6):436–453. 29477613 10.1016/j.tcb.2018.02.001

[48] Miwa S, Kashyap S, Chini E, von Zglinicki T. Mitochondrial dysfunction in cell senescence and aging. J Clin Invest. 2022;132(13): Article e158447. 35775483 10.1172/JCI158447 PMC9246372

[49] LeBrasseur NK, Tchkonia T, Kirkland JL. Cellular senescence and the biology of aging, disease, and frailty. Nestle Nutr Inst Workshop Ser. 2015;83:11–18. 26485647 10.1159/000382054 PMC4780350

[50] Liu J, Wang L, Wang Z, Liu JP. Roles of telomere biology in cell senescence, replicative and chronological ageing. Cells. 2019;8(1):54. 30650660 10.3390/cells8010054 PMC6356700

[51] Zhu J, Wu C, Yang L. Cellular senescence in Alzheimer’s disease: From physiology to pathology. Transl Neurodegener. 2024;13(1):55. 39568081 10.1186/s40035-024-00447-4 PMC11577763

[52] Zhu H, Blake S, Kusuma FK, Pearson RB, Kang J, Chan KT. Oncogene-induced senescence: From biology to therapy. Mech Ageing Dev. 2020;187: Article 111229. 32171687 10.1016/j.mad.2020.111229

[53] Liu XL, Ding J, Meng LH. Oncogene-induced senescence: A double edged sword in cancer. Acta Pharmacol Sin. 2018;39(10):1553–1558. 29620049 10.1038/aps.2017.198 PMC6289471

[54] Prasanna PG, Citrin DE, Hildesheim J, Ahmed MM, Venkatachalam S, Riscuta G, Xi D, Zheng G, Deursen JV, Goronzy J, et al. Therapy-induced senescence: Opportunities to improve anticancer therapy. J Natl Cancer Inst. 2021;113(10):1285–1298. 33792717 10.1093/jnci/djab064 PMC8486333

[55] Regulski MJ. Cellular senescence: What, why, and how. Wounds. 2017;29(6):168–174. 28682291

[56] Salama R, Sadaie M, Hoare M, Narita M. Cellular senescence and its effector programs. Genes Dev. 2014;28(2):99–114. 24449267 10.1101/gad.235184.113 PMC3909793

[57] Wechter N, Rossi M, Anerillas C, Tsitsipatis D, Piao Y, Fan J, Martindale JL, De S, Mazan-Mamczarz K, Gorospe M. Single-cell transcriptomic analysis uncovers diverse and dynamic senescent cell populations. Aging. 2023;15(8):2824–2851. 37086265 10.18632/aging.204666 PMC10188353

[58] Chen MS, Lee RT, Garbern JC. Senescence mechanisms and targets in the heart. Cardiovasc Res. 2022;118(5):1173–1187. 33963378 10.1093/cvr/cvab161 PMC8953446

[59] Basatemur GL, Jørgensen HF, Clarke MCH, Bennett MR, Mallat Z. Vascular smooth muscle cells in atherosclerosis. Nat Rev Cardiol. 2019;16(12):727–744. 31243391 10.1038/s41569-019-0227-9

[60] Gardner SE, Humphry M, Bennett MR, Clarke MC. Senescent vascular smooth muscle cells drive inflammation through an interleukin-1α-dependent senescence-associated secretory phenotype. Arterioscler Thromb Vasc Biol. 2015;35(9):1963–1974. 26139463 10.1161/ATVBAHA.115.305896 PMC4548545

[61] Yao C, Guan X, Carraro G, Parimon T, Liu X, Huang G, Mulay A, Soukiasian HJ, David G, Weigt SS, et al. Senescence of alveolar type 2 cells drives progressive pulmonary fibrosis. Am J Respir Crit Care Med. 2021;203(6):707–717. 32991815 10.1164/rccm.202004-1274OC PMC7958503

[62] Higashi T, Friedman SL, Hoshida Y. Hepatic stellate cells as key target in liver fibrosis. Adv Drug Deliv Rev. 2017;121:27–42. 28506744 10.1016/j.addr.2017.05.007 PMC5682243

[63] Dewidar B, Meyer C, Dooley S, Meindl-Beinker AN. TGF-β in hepatic stellate cell activation and liver fibrogenesis—Updated 2019. Cells. 2019;8(11):1419. 31718044 10.3390/cells8111419 PMC6912224

[64] Todd AC, Hardingham GE. The regulation of astrocytic glutamate transporters in health and neurodegenerative diseases. Int J Mol Sci. 2020;21(24):9607. 33348528 10.3390/ijms21249607 PMC7766851

[65] Baker DJ, Arany Z, Baur JA, Epstein JA, June CH. CAR T therapy beyond cancer: The evolution of a living drug. Nature. 2023;619(7971):707–715. 37495877 10.1038/s41586-023-06243-w PMC12522170

[66] Ellis GI, Sheppard NC, Riley JL. Genetic engineering of T cells for immunotherapy. Nat Rev Genet. 2021;22(7):427–447. 33603158 10.1038/s41576-021-00329-9 PMC8217325

[67] Krause A, Guo HF, Latouche JB, Tan C, Cheung NK, Sadelain M. Antigen-dependent CD28 signaling selectively enhances survival and proliferation in genetically modified activated human primary T lymphocytes. J Exp Med. 1998;188(4):619–626. 9705944 10.1084/jem.188.4.619 PMC2213361

[68] Cherkassky L, Morello A, Villena-Vargas J, Feng Y, Dimitrov DS, Jones DR, Sadelain M, Adusumilli PS. Human CAR T cells with cell-intrinsic PD-1 checkpoint blockade resist tumor-mediated inhibition. J Clin Invest. 2016;126(8):3130–3144. 27454297 10.1172/JCI83092 PMC4966328

[69] Rafiq S, Yeku OO, Jackson HJ, Purdon TJ, van Leeuwen DG, Drakes DJ, Song M, Miele MM, Li Z, Wang P, et al. Targeted delivery of a PD-1-blocking scFv by CAR-T cells enhances anti-tumor efficacy in vivo. Nat Biotechnol. 2018;36(9):847–856. 30102295 10.1038/nbt.4195 PMC6126939

[70] Agarwal S, Aznar MA, Rech AJ, Good CR, Kuramitsu S, Da T, Gohil M, Chen L, Hong SJ, Ravikumar P, et al. Deletion of the inhibitory co-receptor CTLA-4 enhances and invigorates chimeric antigen receptor T cells. Immunity. 2023;56(10):2388–2407.e9. 37776850 10.1016/j.immuni.2023.09.001 PMC10591801

[71] Xiao W, Xu L, Wang J, Yu K, Xu B, Que Y, Zhao J, Pan Q, Gao C, Zhou P, et al. FGFR4-specific CAR-T cells with inducible caspase-9 suicide gene as an approach to treat rhabdomyosarcoma. Cancer Gene Ther. 2024;31(10):1571–1584. 39183354 10.1038/s41417-024-00823-2 PMC11489081

[72] Liu L, Wang Y. EchoBack-CAR T cells: Tuning immunity with sound. Clin Transl Med. 2025;15(7): Article e70391. 10.1002/ctm2.70391 PMC12211968 40591245

[73] Liu L, He P, Wang Y, Ma F, Li D, Bai Z, Qu Y, Zhu L, Yoon CW, Yu X, et al. Engineering sonogenetic EchoBack-CAR T cells. Cell. 2025;188(10):2621–2636.e20. 40179881 10.1016/j.cell.2025.02.035 PMC12085297

[74] McKee B, Liu S, Cai PX, Yang Z, Lan TH, Zhou Y. Optogenetic control of T cells for immunomodulation. Essays Biochem. 2025;69(2):33–46. 40548402 10.1042/EBC20253014 PMC12228418

[75] Zhao Y, Li R, Han Y, Shi C, Lee K, Nie G, Chen Y. On-demand cancer immunotherapy via single-cell encapsulation of synthetic circuit-engineered cells. Sci Adv. 2026;12(3): Article eaea3573. 41533781 10.1126/sciadv.aea3573 PMC12802821

[76] Jetley U, Balwani I, Sharma P, Miller IC, Luther A, Dutta I, Saravanan N, Goel S, Zhang Q, Zhang B, et al. A differentiated and durable allogeneic strategy applicable to cell therapies. Cytotherapy. 2026;28(3): Article 101991. 41201433 10.1016/j.jcyt.2025.10.001

[77] Henschel P, Landwehr-Kenzel S, Engels N, Schienke A, Kremer J, Riet T, Redel N, Iordanidis K, Saetzler V, John K, et al. Supraphysiological FOXP3 expression in human CAR-Tregs results in improved stability, efficacy, and safety of CAR-Treg products for clinical application. J Autoimmun. 2023;138: Article 103057. 37224732 10.1016/j.jaut.2023.103057

[78] Niu C, Wei H, Pan X, Wang Y, Song H, Li C, Qie J, Qian J, Mo S, Zheng W, et al. Foxp3 confers long-term efficacy of chimeric antigen receptor-T cells via metabolic reprogramming. Cell Metab. 2025;37(6):1426–1441.e7. 40328248 10.1016/j.cmet.2025.04.008

[79] Zhao Z, Sadelain M. CAR T cell design: Approaching the elusive AND-gate. Cell Res. 2023;33(10):739–740. 37221269 10.1038/s41422-023-00828-w PMC10542763

[80] von Jutrzenka-Trzebiatowski A, Gupte R, Daglar C, Berndt N, Arndt C, Bachmann M, Feldmann A. CliniMACS prodigy manufacturing of switchable, AND-gate CAR T cells. Int J Mol Sci. 2025;26(11): Article 5024. 40507834 10.3390/ijms26115024 PMC12154027

[81] Khanali J, Azangou-Khyavy M, Boroomand-Saboor M, Ghasemi M, Niknejad H. JAK/STAT-dependent chimeric antigen receptor (CAR) expression: A design benefiting from a dual AND/OR gate aiming to increase specificity, reduce tumor escape and affect tumor microenvironment. Front Immunol. 2021;12: Article 638639. 34177890 10.3389/fimmu.2021.638639 PMC8220211

[82] Mahmoud MM, Abdel Hamid FF, Abdelgawad I, Ismail A, Malash I, Ibrahim DM. Diagnostic efficacy of PSMA and PSCA mRNAs combined to PSA in prostate cancer patients. Asian Pac J Cancer Prev. 2023;24(1):223–229. 36708571 10.31557/APJCP.2023.24.1.223 PMC10152839

[83] Wen J, Wu L, Zhong S, Shan H, Luo JL. The role of GPI-anchored LY6/uPAR family proteins in connecting membrane microdomains with immune regulation and diseases. Crit Rev Oncol Hematol. 2025;216: Article 104971. 41016505 10.1016/j.critrevonc.2025.104971

[84] Huang Y, Liu T. Step further towards targeted senolytic therapy: Therapeutic potential of uPAR-CAR T cells for senescence-related diseases. Signal Transduct Target Ther. 2020;5(1):155. 32792494 10.1038/s41392-020-00268-7 PMC7426266

[85] Asahina M, Yoshiyama Y, Hattori T. Expression of matrix metalloproteinase-9 and urinary-type plasminogen activator in Alzheimer’s disease brain. Clin Neuropathol. 2001;20(2):60–63. 11327298

[86] Li JH, Chen YY. A fresh approach to targeting aging cells: CAR-T cells enhance senolytic specificity. Cell Stem Cell. 2020;27(2):192–194. 32763179 10.1016/j.stem.2020.07.010 PMC7717659

[87] Amor C, Feucht J, Leibold J, Ho YJ, Zhu C, Alonso-Curbelo D, Mansilla-Soto J, Boyer JA, Li X, Giavridis T, et al. Senolytic CAR T cells reverse senescence-associated pathologies. Nature. 2020;583(7814):127–132. 32555459 10.1038/s41586-020-2403-9 PMC7583560

[88] Amor C, Fernández-Maestre I, Chowdhury S, Ho YJ, Nadella S, Graham C, Carrasco SE, Nnuji-John E, Feucht J, Hinterleitner C, et al. Prophylactic and long-lasting efficacy of senolytic CAR T cells against age-related metabolic dysfunction. Nat Aging. 2024;4(3):336–349. 38267706 10.1038/s43587-023-00560-5 PMC10950785

[89] Yashaswini CN, Qin T, Bhattacharya D, Amor C, Lowe S, Lujambio A, Wang S, Friedman SL. Phenotypes and ontogeny of senescent hepatic stellate cells in metabolic dysfunction-associated steatohepatitis. J Hepatol. 2024;81(2):207–217. 38508241 10.1016/j.jhep.2024.03.014 PMC11269047

[90] Zhang Z, Ma B, Li B, Li Z, Gao M, Zhao H, Peng R, Hu J, Wang Y, You W, et al. Cardiolipin-mimic lipid nanoparticles without antibody modification delivered senolytic in vivo CAR-T therapy for inflammaging. Cell Rep Med. 2025;6(7): Article 102209. 40602406 10.1016/j.xcrm.2025.102209 PMC12281384

[91] Sagiv A, Burton DG, Moshayev Z, Vadai E, Wensveen F, Ben-Dor S, Golani O, Polic B, Krizhanovsky V. NKG2D ligands mediate immunosurveillance of senescent cells. Aging. 2016;8(2):328–344. 26878797 10.18632/aging.100897 PMC4789586

[92] Yang D, Sun B, Li S, Wei W, Liu X, Cui X, Zhang X, Liu N, Yan L, Deng Y, et al. NKG2D-CAR T cells eliminate senescent cells in aged mice and nonhuman primates. Sci Transl Med. 2023;15(709): Article eadd1951. 37585504 10.1126/scitranslmed.add1951

[93] Obajdin J, Larcombe-Young D, Glover M, Kausar F, Hull CM, Flaherty KR, Tan G, Beatson RE, Dunbar P, Mazza R, et al. Solid tumor immunotherapy using NKG2D-based adaptor CAR T cells. Cell Rep Med. 2024;5(11): Article 101827. 39566469 10.1016/j.xcrm.2024.101827 PMC11604534

[94] Deng Y, Kumar A, Xie K, Schaaf K, Scifo E, Morsy S, Li T, Ehninger A, Bano D, Ehninger D. Targeting senescent cells with NKG2D-CAR T cells. Cell Death Discov. 2024;10(1):217. 38704364 10.1038/s41420-024-01976-7 PMC11069534

[95] Zhao S, Li R, Xia Y, Wang X, Liu Z, Chu Q, He J, Zhang J, Guo Y, Wang Y, et al. Targeting ECM-producing cells with CAR-T therapy alleviates fibrosis in chronic kidney disease. Cell Stem Cell. 2025;32(9):1390–1402.e9. 40848726 10.1016/j.stem.2025.07.014

[96] Aghajanian H, Kimura T, Rurik JG, Hancock AS, Leibowitz MS, Li L, Scholler J, Monslow J, Lo A, Han W, et al. Targeting cardiac fibrosis with engineered T cells. Nature. 2019;573(7774):430–433. 31511695 10.1038/s41586-019-1546-z PMC6752964

[97] Boskovic P, Shalita R, Gao W, Vernon H, Deng YL, Colonna M, Majzner RG, Amit I, Kipnis J. Engineering chimeric antigen receptor CD4 T cells for Alzheimer’s disease. Proc Natl Acad Sci USA. 2026;123(7): Article e2530977123. 41662521 10.1073/pnas.2530977123 PMC12912999

[98] Meca-Laguna G, Qiu M, Hou Y, Barkovskaya A, Shankar A, Dixit B, Rae MJ, Boominathan A, Sharma A. Cell-surface LAMP1 is a senescence marker in aging and idiopathic pulmonary fibrosis. Aging Cell. 2025;24(9): Article e70141. 40545776 10.1111/acel.70141 PMC12419843

[99] Jha R, Shi J, Sedgwick MJ, Sui J, Corcoran TE, Kliment CR. Reduced mitochondrial adenine nucleotide translocase 1 (ANT1) correlates with aging-associated airway remodeling. Aging Cell. 2025;24(12): Article e70264. 41074556 10.1111/acel.70264 PMC12686547

[100] Madsen SD, Russell KC, Tucker HA, Glowacki J, Bunnell BA, O’Connor KC. Decoy TRAIL receptor CD264: A cell surface marker of cellular aging for human bone marrow-derived mesenchymal stem cells. Stem Cell Res Ther. 2017;8(1):201. 28962588 10.1186/s13287-017-0649-4 PMC5622446

[101] Chen R, Zhang X, Chen J. KAT8 knockdown reverses hucMSC senescence and enhances diabetic wound healing efficacy. Exp Clin Endocrinol Diabetes. 2025;133(9):441–452. 40816295 10.1055/a-2684-5975

[102] Chen YH, Zhang X, Chou CH, Hsueh MF, Attarian D, Li YJ, Kraus VB. Association of dipeptidylpeptidase 4 (CD26) with chondrocyte senescence and radiographic progression in knee osteoarthritis. Arthritis Rheumatol. 2023;75(7):1120–1131. 36704903 10.1002/art.42455 PMC10313751

[103] Fissoun C, Maroun G, Silva R, Milano M, Guibert B, Dagneaux L, Ferreira-Lopez R, Commes T, Gilson E, Jorgensen C, et al. The ganglioside GD3 and its synthase (ST8SIA1) as novel senescence markers associated with osteoarthritis. GeroScience. 2026;48(2):2013–2027. 41102476 10.1007/s11357-025-01903-3 PMC12972427

[104] Rule AD, Amer H, Cornell LD, Taler SJ, Cosio FG, Kremers WK, Textor SC, Stegall MD. The association between age and nephrosclerosis on renal biopsy among healthy adults. Ann Intern Med. 2010;152(9):561–567. 20439574 10.1059/0003-4819-152-9-201005040-00006 PMC2864956

[105] Luo C, Zhou S, Zhou Z, Liu Y, Yang L, Liu J, Zhang Y, Li H, Liu Y, Hou FF, et al. Wnt9a promotes renal fibrosis by accelerating cellular senescence in tubular epithelial cells. J Am Soc Nephrol. 2018;29(4):1238–1256. 29440280 10.1681/ASN.2017050574 PMC5875944

[106] Anderson R, Lagnado A, Maggiorani D, Walaszczyk A, Dookun E, Chapman J, Birch J, Salmonowicz H, Ogrodnik M, Jurk D, et al. Length-independent telomere damage drives post-mitotic cardiomyocyte senescence. EMBO J. 2019;38(5): Article e100492. 30737259 10.15252/embj.2018100492 PMC6396144

[107] Kurundkar A, Thannickal VJ. Redox mechanisms in age-related lung fibrosis. Redox Biol. 2016;9:67–76. 27394680 10.1016/j.redox.2016.06.005 PMC4943089

[108] Schafer MJ, White TA, Iijima K, Haak AJ, Ligresti G, Atkinson EJ, Oberg AL, Birch J, Salmonowicz H, Zhu Y, et al. Cellular senescence mediates fibrotic pulmonary disease. Nat Commun. 2017;8(1):14532. 28230051 10.1038/ncomms14532 PMC5331226

[109] Guo Y, Wu S, Ye W, Zhao Z, Li K, Guo X, Chen W, Cai S, Zhan M, Huang Z, et al. Impact of public health and social measures on contact dynamics during a SARS-CoV-2 Omicron variant outbreak in Quanzhou, China, March to April 2022. Int J Infect Dis. 2023;131:46–49. 36967039 10.1016/j.ijid.2023.03.025 PMC10037913

[110] Khalil H, Kanisicak O, Prasad V, Correll RN, Fu X, Schips T, Vagnozzi RJ, Liu R, Huynh T, Lee SJ, et al. Fibroblast-specific TGF-β-Smad2/3 signaling underlies cardiac fibrosis. J Clin Invest. 2017;127(10):3770–3783. 28891814 10.1172/JCI94753 PMC5617658

[111] Trinh-Minh T, Chen CW, Tran Manh C, Li YN, Zhu H, Zhou X, Chakraborty D, Zhang Y, Rauber S, Dees C, et al. Noncanonical WNT5A controls the activation of latent TGF-β to drive fibroblast activation and tissue fibrosis. J Clin Invest. 2024;134(10): Article e159884. 38747285 10.1172/JCI159884 PMC11093613

[112] Takamura N, Renaud L, da Silveira WA, Feghali-Bostwick C. PDGF promotes dermal fibroblast activation via a novel mechanism mediated by signaling through MCHR1. Front Immunol. 2021;12: Article 745308. 34912333 10.3389/fimmu.2021.745308 PMC8667318

[113] Yang P, Luo Q, Wang X, Fang Q, Fu Z, Li J, Lai Y, Chen X, Xu X, Peng X, et al. Comprehensive analysis of fibroblast activation protein expression in interstitial lung diseases. Am J Respir Crit Care Med. 2023;207(2):160–172. 35984444 10.1164/rccm.202110-2414OC PMC9893314

[114] Fitzgerald AA, Weiner LM. The role of fibroblast activation protein in health and malignancy. Cancer Metastasis Rev. 2020;39(3):783–803. 32601975 10.1007/s10555-020-09909-3 PMC7487063

[115] Rurik JG, Tombácz I, Yadegari A, Méndez Fernández PO, Shewale SV, Li L, Kimura T, Soliman OY, Papp TE, Tam YK, et al. CAR T cells produced in vivo to treat cardiac injury. Science. 2022;375(6576):91–96. 34990237 10.1126/science.abm0594 PMC9983611

[116] Hickson LJ, Langhi Prata LGP, Bobart SA, Evans TK, Giorgadze N, Hashmi SK, Herrmann SM, Jensen MD, Jia Q, Jordan KL, et al. Senolytics decrease senescent cells in humans: Preliminary report from a clinical trial of dasatinib plus quercetin in individuals with diabetic kidney disease. EBioMedicine. 2019;47:446–456. 31542391 10.1016/j.ebiom.2019.08.069 PMC6796530

[117] Xu M, Pirtskhalava T, Farr JN, Weigand BM, Palmer AK, Weivoda MM, Inman CL, Ogrodnik MB, Hachfeld CM, Fraser DG, et al. Senolytics improve physical function and increase lifespan in old age. Nat Med. 2018;24(8):1246–1256. 29988130 10.1038/s41591-018-0092-9 PMC6082705

[118] Hickson LJ, Langhi Prata LGP, Bobart SA, Evans TK, Giorgadze N, Hashmi SK, Herrmann SM, Jensen MD, Jia Q, Jordan KL, et al. Corrigendum to ‘Senolytics decrease senescent cells in humans: Preliminary report from a clinical trial of dasatinib plus quercetin in individuals with diabetic kidney disease’ EBioMedicine 47 (2019) 446-456. EBioMedicine. 2020;52: Article 102595. 10.1016/j.ebiom.2019.08.069 PMC6796530 31542391

[119] Justice JN, Nambiar AM, Tchkonia T, LeBrasseur NK, Pascual R, Hashmi SK, Prata L, Masternak MM, Kritchevsky SB, Musi N, et al. Senolytics in idiopathic pulmonary fibrosis: Results from a first-in-human, open-label, pilot study. EBioMedicine. 2019;40:554–563. 30616998 10.1016/j.ebiom.2018.12.052 PMC6412088

[120] Smer-Barreto V, Quintanilla A, Elliott RJR, Dawson JC, Sun J, Campa VM, Lorente-Macías Á, Unciti-Broceta A, Carragher NO, Acosta JC, et al. Discovery of senolytics using machine learning. Nat Commun. 2023;14(1):3445. 37301862 10.1038/s41467-023-39120-1 PMC10257182

[121] Rad AN, Grillari J. Current senolytics: Mode of action, efficacy and limitations, and their future. Mech Ageing Dev. 2024;217: Article 111888. 38040344 10.1016/j.mad.2023.111888

[122] Robbins PD, Jurk D, Khosla S, Kirkland JL, LeBrasseur NK, Miller JD, Passos JF, Pignolo RJ, Tchkonia T, Niedernhofer LJ. Senolytic drugs: Reducing senescent cell viability to extend health span. Annu Rev Pharmacol Toxicol. 2021;61(1):779–803. 32997601 10.1146/annurev-pharmtox-050120-105018 PMC7790861

[123] Zhu Y, Ge J, Huang C, Liu H, Jiang H. Application of mesenchymal stem cell therapy for aging frailty: From mechanisms to therapeutics. Theranostics. 2021;11(12):5675–5685. 33897874 10.7150/thno.46436 PMC8058725

[124] Raggi C, Berardi AC. Mesenchymal stem cells, aging and regenerative medicine. Muscles Ligaments Tendons J. 2012;2(3):239–242. 23738303 PMC3666525

[125] Lee BY, Han JA, Im JS, Morrone A, Johung K, Goodwin EC, Kleijer WJ, DiMaio D, Hwang ES. Senescence-associated β-galactosidase is lysosomal β-galactosidase. Aging Cell. 2006;5(2):187–195. 16626397 10.1111/j.1474-9726.2006.00199.x

[126] Calcinotto A, Kohli J, Zagato E, Pellegrini L, Demaria M, Alimonti A. Cellular senescence: Aging, cancer, and injury. Physiol Rev. 2019;99(2):1047–1078. 30648461 10.1152/physrev.00020.2018

[127] Lee AJ, Kim C, Park S, Joo J, Choi B, Yang D, Jun K, Eom J, Lee SJ, Chung SJ, et al. Characterization of altered molecular mechanisms in Parkinson’s disease through cell type-resolved multiomics analyses. Sci Adv. 2023;9(15): Article eabo2467. 37058563 10.1126/sciadv.abo2467 PMC10104466

[128] Zah E, Lin MY, Silva-Benedict A, Jensen MC, Chen YY. T cells expressing CD19/CD20 bispecific chimeric antigen receptors prevent antigen escape by malignant B cells. Cancer Immunol Res. 2016;4(6):498–508. 27059623 10.1158/2326-6066.CIR-15-0231 PMC4933590

[129] Hegde M, Mukherjee M, Grada Z, Pignata A, Landi D, Navai SA, Wakefield A, Fousek K, Bielamowicz K, Chow KK, et al. Tandem CAR T cells targeting HER2 and IL13Rα2 mitigate tumor antigen escape. J Clin Invest. 2016;126(8):3036–3052. 27427982 10.1172/JCI83416 PMC4966331

[130] Ferrucci L, Fabbri E. Inflammageing: Chronic inflammation in ageing, cardiovascular disease, and frailty. Nat Rev Cardiol. 2018;15(9):505–522. 30065258 10.1038/s41569-018-0064-2 PMC6146930

[131] Labanieh L, Majzner RG, Klysz D, Sotillo E, Fisher CJ, Vilches-Moure JG, Pacheco KZ, Malipatlolla M, Xu P, Hui JH, et al. Enhanced safety and efficacy of protease-regulated CAR-T cell receptors. Cell. 2022;185(10):1745–1763.e22. 35483375 10.1016/j.cell.2022.03.041 PMC9467936

[132] Gargett T, Brown MP. The inducible caspase-9 suicide gene system as a “safety switch” to limit on-target, off-tumor toxicities of chimeric antigen receptor T cells. Front Pharmacol. 2014;5:235. 25389405 10.3389/fphar.2014.00235 PMC4211380

[133] Warda W, Da Rocha MN, Trad R, Haderbache R, Salma Y, Bouquet L, Roussel X, Nicod C, Deschamps M, Ferrand C. Overcoming target epitope masking resistance that can occur on low-antigen-expresser AML blasts after IL-1RAP chimeric antigen receptor T cell therapy using the inducible caspase 9 suicide gene safety switch. Cancer Gene Ther. 2021;28(12):1365–1375. 33414517 10.1038/s41417-020-00284-3 PMC8636256

[134] Jones BS, Lamb LS, Goldman F, Di Stasi A. Improving the safety of cell therapy products by suicide gene transfer. Front Pharmacol. 2014;5:254. 25505885 10.3389/fphar.2014.00254 PMC4245885

[135] Tao Z, Chyra Z, Kotulová J, Celichowski P, Mihályová J, Charvátová S, Hájek R. Impact of T cell characteristics on CAR-T cell therapy in hematological malignancies. Blood Cancer J. 2024;14(1):213. 39627220 10.1038/s41408-024-01193-6 PMC11615218

[136] Nikolich-Žugich J. Ageing and life-long maintenance of T-cell subsets in the face of latent persistent infections. Nat Rev Immunol. 2008;8(7):512–522. 18469829 10.1038/nri2318 PMC5573867

[137] Fu Y, Wang B, Alu A, Hong W, Lei H, He X, Shi H, Cheng P, Yang X. Immunosenescence: Signaling pathways, diseases and therapeutic targets. Signal Transduct Target Ther. 2025;10(1):250. 40769978 10.1038/s41392-025-02371-z PMC12328749

[138] Park JH, Lee SW, Choi D, Lee C, Sung YC. Harnessing the power of IL-7 to boost T cell immunity in experimental and clinical immunotherapies. Immune Netw. 2024;24(1): Article e9. 38455462 10.4110/in.2024.24.e9 PMC10917577

[139] Han S, Georgiev P, Ringel AE, Sharpe AH, Haigis MC. Age-associated remodeling of T cell immunity and metabolism. Cell Metab. 2023;35(1):36–55. 36473467 10.1016/j.cmet.2022.11.005 PMC10799654

[140] Bai Y, Kan S, Zhou S, Wang Y, Xu J, Cooke JP, Wen J, Deng H. Enhancement of the in vivo persistence and antitumor efficacy of CD19 chimeric antigen receptor T cells through the delivery of modified TERT mRNA. Cell Discov. 2015;1(1):15040. 27462436 10.1038/celldisc.2015.40 PMC4860832

[141] Onorati A, Havas AP, Lin B, Rajagopal J, Sen P, Adams PD, Dou Z. Upregulation of PD-L1 in senescence and aging. Mol Cell Biol. 2022;42(10): Article e0017122. 36154662 10.1128/mcb.00171-22 PMC9583718

[142] Sui BD, Hu CH, Zheng CX, Jin Y. Microenvironmental views on mesenchymal stem cell differentiation in aging. J Dent Res. 2016;95(12):1333–1340. 27302881 10.1177/0022034516653589

[143] Gao Y, Chi Y, Chen Y, Wang W, Li H, Zheng W, Zhu P, An J, Duan Y, Sun T, et al. Multi-omics analysis of human mesenchymal stem cells shows cell aging that alters immunomodulatory activity through the downregulation of PD-L1. Nat Commun. 2023;14(1):4373. 37474525 10.1038/s41467-023-39958-5 PMC10359415

[144] Wang X, Luo W, Chen Z, Li C, Zhou J, Huang Z, Tang L, Wu J, Wu Z, Li Y, et al. Co-expression of IL-15 and CCL21 strengthens CAR-NK cells to eliminate tumors in concert with T cells and equips them with PI3K/AKT/mTOR signal signature. J Immunother Cancer. 2025;13(6): Article e010822. 40518288 10.1136/jitc-2024-010822 PMC12314829

[145] Steffin D, Ghatwai N, Montalbano A, Rathi P, Courtney AN, Arnett AB, Fleurence J, Sweidan R, Wang T, Zhang H, et al. Interleukin-15-armoured GPC3 CAR T cells for patients with solid cancers. Nature. 2025;637(8047):940–946. 39604730 10.1038/s41586-024-08261-8 PMC12704925

[146] Frede J, Poller JC, Shi K, Stuart H, Sotudeh N, Havig C, Lim K, Wiggers CR, Cho EY, Vijaykumar T, et al. The endogenous T cell landscape is reshaped by CAR-T cell therapy and predicts treatment response in multiple myeloma. Leukemia. 2025;39(12):3004–3014. 40973768 10.1038/s41375-025-02766-5 PMC12634420

[147] Cai N, Wu Y, Huang Y. Induction of accelerated aging in a mouse model. Cells. 2022;11(9): Article 1418. 35563724 10.3390/cells11091418 PMC9102583

[148] Mori M, Higuchi K. The senescence-accelerated mouse as a model for geriatrics and aging biology. Nihon Yakurigaku Zasshi. 2019;153(4):179–185. 30971658 10.1254/fpj.153.179

[149] Lu J, Ahmad R, Nguyen T, Cifello J, Hemani H, Li J, Chen J, Li S, Wang J, Achour A, et al. Heterogeneity and transcriptome changes of human CD8^+^ T cells across nine decades of life. Nat Commun. 2022;13(1):5128. 36050300 10.1038/s41467-022-32869-x PMC9436929

[150] Meng D, Zhang S, Huang Y, Mao K, Han J-DJ. Application of AI in biological age prediction. Curr Opin Struct Biol. 2024;85: Article 102777. 38310737 10.1016/j.sbi.2024.102777

[151] Qiu S, Chen J, Wu T, Li L, Wang G, Wu H, Song X, Liu X, Wang H. CAR-Toner: An AI-driven approach for CAR tonic signaling prediction and optimization. Cell Res. 2024;34(5):386–388. 38351128 10.1038/s41422-024-00936-1 PMC11061301

[152] Eskiocak O, Chowdhury S, Shah V, Nnuji-John E, Chung C, Boyer JA, Harris AS, Habel J, Sadelain M, Beyaz S, et al. Senolytic CAR T cells reverse aging-associated defects in intestinal regeneration and fitness. bioRxiv. 2024. 10.1101/2024.03.19.585779.

[153] Vagnozzi RJ, Johansen AKZ, Molkentin JD. CARdiac immunotherapy: T cells engineered to treat the fibrotic heart. Mol Ther. 2019;27(11):1869–1871. 31585799 10.1016/j.ymthe.2019.09.021 PMC6838878

[154] Buhl EM, Djudjaj S, Klinkhammer BM, Ermert K, Puelles VG, Lindenmeyer MT, Cohen CD, He C, Borkham-Kamphorst E, Weiskirchen R, et al. Dysregulated mesenchymal PDGFR-β drives kidney fibrosis. EMBO Mol Med. 2020;12(3): Article e11021. 31943786 10.15252/emmm.201911021 PMC7059015

[155] Xu J, Lin SC, Chen J, Miao Y, Taffet GE, Entman ML, Wang Y. CCR2 mediates the uptake of bone marrow-derived fibroblast precursors in angiotensin II-induced cardiac fibrosis. Am J Physiol Heart Circ Physiol. 2011;301(2):H538–H547. 21572015 10.1152/ajpheart.01114.2010 PMC3154672

[156] Frangogiannis NG, Entman ML. Chemokines in myocardial ischemia. Trends Cardiovasc Med. 2005;15(5):163–169. 16165012 10.1016/j.tcm.2005.06.005

[157] Rossi M, Abdelmohsen K. The emergence of senescent surface biomarkers as Senotherapeutic targets. Cells. 2021;10(7): Article 1740. 34359910 10.3390/cells10071740 PMC8305747

[158] Wang Y, Xie F, He Z, Che L, Chen X, Yuan Y, Liu C. Senescence-targeted and NAD^+^-dependent SIRT1-activated Nanoplatform to counteract stem cell senescence for promoting aged bone regeneration. Small. 2024;20(12): Article e2304433. 37948437 10.1002/smll.202304433

