# Supplementary material for: Chimeric Antigen Receptor T Cells as Living Therapeutics Targeting Senescence and Age-Related Diseases
Source: Research (Wash D C). 2026 Jun 12;9:1326. doi: 10.34133/research.1326 (PMC13260815; doi:10.34133/research.1326)
Supplement: Supplementary 1 — Table S1 Reference [158] [file research.1326.f1.docx]

**Table S1 New Targets for senescent cells**

| **Target** | **Organs/Tissues/Cells** | **Main preclinical evidence** | **Clinical potential** | **Target Localization** |
| --- | --- | --- | --- | --- |
| LAMP1[98] | Fibrotic lung | In human and mouse senescent cells, LAMP1 is selectively upregulated; cells enriched for Lamp1 coexpress multiple canonical senescence markers. | It holds considerable potential for treating pulmonary fibrosis-related disorders, while research in the context of CAR-T therapy remains limited. | Cell surface / Plasma membrane |
| ANT1[99] | Fibrotic lung | The knockout of Ant1 in mice leads to an increase in aging markers; knockdown of Ant1 in airway epithelial cells results in the upregulation of genes related to aging and tissue remodeling. | It has only been studied in mouse models and has not yet entered the clinical research stage. | Intracellular: Mitochondria |
| CD264[100] | Mesenchymal stem cells(MSCs) | CD264 is initially upregulated in MSCs during the intermediate stage of cellular senescence and remains elevated throughout the aging process. | Low clinical potential | Cell surface / Plasma membrane |
| KAT8[101] | Human umbilical cord mesenchymal stem cells(hucMSCs) | During the senescence of human mesenchymal stem cells, KAT8 expression decreases. Knockdown of KAT8 downregulates senescence-associated genes (such as P21 and P16), while enhancing the proliferation, migration, and survival of hucMSCs without altering the expression of surface stem cell markers. | Only validation was conducted on hucMSC, and no animal or clinical trials were carried out. | Intracellular: Nuclear |
| Kremen1[159] | Mesenchymal stem cells(MSCs) | A novel MSCs-specific senescence surface marker, combining with its antibody, enhances the affinity of nanomedicines for senescent mesenchymal stem cells. | For the validation of the target sites on the cells, there are currently no clinical trials involving CAR-T. | Cell surface / Plasma membrane |
| GD3[103] | Osteocyte | GD3 is a senescence-associated cell surface marker. The expression of GD3 synthase, ST8SIA1, is increased in patients with osteoarthritis, and GD3-positive human osteoarthritic chondrocytes or synovial cells are enriched for senescence and SASP markers. | It has only been studied at the preclinical stage and has not yet entered the clinical research phase. | Cell surface / Plasma membrane |
